# Acknowledgement to Reviewers of *IJERPH* in 2018

**DOI:** 10.3390/ijerph16010160

**Published:** 2019-01-08

**Authors:** IJERPH Editorial Office

**Affiliations:** MDPI, St. Alban-Anlage 66, 4052 Basel, Switzerland

Rigorous peer-review is the corner-stone of high-quality academic publishing. The editorial team greatly appreciates the reviewers who contributed their knowledge and expertise to the journal’s editorial process over the past 12 months. In 2018, a total of 2910 papers were published in the journal, with a median time to first decision of 19 days and a median time to publication of 43 days. The editors would like to express their sincere gratitude to the following reviewers for their cooperation and dedication in 2018:
Aadland, EivindLi, YangAarabi, MahmoudLi, ZhianAbatih, EmmanuelLi, JianbinAbbas, AzharLi, ChunxiaoAbbod, MaysamLi, XinAbdelghani, AssafLi, XiuyangAbdelhamid, Tariq SamiLi, XianjuAbdelkarim, OsamaLi, BingqinAbecasis, FranciscoLi, SenAbeysuriya, RomeshLi, XiaopingAbidi, SaminaLi, YuanyuanAbraham, John PLi, LianboAbrahamson, VanessaLi, ZhonggenAbrams, DavidLi, XiaAbreu, WilsonLi, An-DongAbreu, AntonioLi, YanAbritis, AlisonLi, XiaofangAbyaneh, Majid KazemianLi, Shao-GangAccardi, RositaLiang, HongAccinelli, CesareLiang, YanAccoroni, StefanoLiang, HengAcevedo, Raquel MayordomoLiang, TiebingAcevedo, Rubén CarlosLiang, YutingAchterberg, Eric P.Liang, JianmingAcquadro Maran, DanielaLiang, JieAdachi, TakeyaLiao, Chien-SenAdamiec, EwaLiao, Yi-JenAdams, MatthewLiao, YungAdams, Katherine P.Liao, MiaoAdamus, GrażynaLiao, Jyh-feiAdeleye, AdeyemiLiao, Yao-ShengAdnan Raheel Shah, SyyedLiapi, CharisAdonakis, GeorgeLiaw, Shyue-CherngAdusei-Asante, KwadwoLiberda, Eric N.Aerts, OlivierLibralato, GiovanniAfrianty, DinaLichtveld, KimAgathokleous, EvgeniosLicitra, GaetanoAggarwal, AnjuLidbury, BrettAggazzotti, GabriellaLiddle, Brantley T.Agodi, AntonellaLiese, AngelaAgrawal, PriyankaLievanos, RaoulAgupusi, PatriciaLignier, BaptisteAhern, TraceyLilenfeld, LisaAhi, YadvinderLilja, MargaretaAhlenstiel, ChantelleLiller, KarenAhlfs-Dunn, SarahLilley, RebbeccaAhlstrom, ChristinaLillrank, PaulAhmadi, ShukrullahLin, Shin-JongAhmed, WarishLin, WeiAhmed, ZehraLin, Chyongchiou JengAhn, JunLin, Hsien-ChangAhonen, MerjaLin, Hai-ShuAi, ZhengtaoLin, QianAittasalo, MinnaLin, Yi-ChinAkbar, SaminaLin, Ming-YengAkintobi, Tabia HenryLin, Pei-KuanAkobirshoev, IlhomLin, SainanAkombi, Blessing J.Lin, QiangAkpinar, AbdullahLin, YanAkushevich, Igor V.Lin, Chih-MingAlafifi, AymanLin, Gen-MinAlam, BhuiyanLin, ZhoumengAlam, AshrafulLin, Yu-HsuanAlamaniotis, MiltiadisLin, Ro-TingAlava, Juan JoséLin, HebinAlberdi, PilarLin, I-ChunAlbert, JeffLin, Chin-FengAlbrechtsen, Hans-JørgenLin, Tang-huangAlcock, LisaLin, Yen-KoAldieri, LuigiLin, Pao-HwaAldous, ColleenLin, ShengdaAldrich, Rosalie S.Lind, HansAleluia Da Silva Reis, LaraLindahl, JohannaAletta, FrancescoLindell, MichaelAlexander, NealLinder, Stephen H.Al-Fatimi, MohamedLindsay, KarenAlfonso, MoyaLindsey, Eric W.Al-Gamrh, Bakr AliLing, ChrisAl-Gasseer, NaeemaLing, MauriceAlho, RolleLinks, PaulAli, RaianLiolios, Konstantinos A.Ali, AamerLiotta, GiuseppeAlison, InnerdLiou, Tsan-HonAl-Jawaldeh, AyoubLisa, AntonellaAlkhatib, AhmadLisinskienė, AušraAlkorta, ItziarLissauer, DavidAllegaert, KarelLitofsky, Norman ScottAllem, Jon-PatrickLitschauer, BrigitteAllen, VivietteLiu, ZhaorongAllore, Heather G.Liu, LunAlm, SusanneLiu, Shing-HwaAlmberg, Kirsten StaggsLiu, Cheuk LunAlmeida, AgostinhoLiu, ChengfangAlmeida, Maria Adelaide AraújoLiu, YanbiaoAl-Muqdadi, Sameh W.Liu, TingtingAlomari, KasimLiu, Wen-ChengAlonzo, DanaLiu, JianguoAlsharairi, NaserLiu, NanAlshbool, Fatima Z.Liu, RenzhiAlt, David PLiu, XingpengAltan, HasimLiu, WeiboAltenburg, TeatskeLiu, GeorgeAlvarez, José DomingoLiu, WenbinÁlvarez-García, JoséLiu, Hu-chenAlves, ElisabeteLiu, JianfengAlves, JulianaLiu, YanAlves-Pereira, MarianaLiu, WenAmbroszkiewicz, JadwigaLiu, ShaoqingAmer, HayderLiu, BianAmetzaga, IboneLiu, WeiAmini, RezaLiu, JieminAmini, Rose MarieLiu, Chen-YuAmini, HereshLiu, CaixiaAmirkhani, MasoumeLiu, RebeccaAmmer, KurtLiu, JianlinAmorena, MicheleLizundia, ErlantzAn, ChunjiangLloret, AntonioAnadón, ArturoLloyd, VettAnafi, PatriciaLloyd, NatalieAnanga, ErickLloyd-Sherlock, PeterAnastasio, PhyllisLo, Huang-MuAncona, CarlaLo, Wei-ShuoAnderberg, PeterLo, Yu-ShengAnderson, CraigLo, EuniceAndina-Díaz, ElenaLo Coco, GianlucaAndrade, Vanda Maria Falcão Espada Lopes DeLoáiciga, Hugo A.Andrasik, MicheleLochbaum, MarcAndreas, SeidlerLockett, HelenAndreassen, MonicaLockie, RobertAndreassi, MariaLodovici, MauraAndreoli, EnricoLofts, StephenAndrews-Speed, PhilipLoftus, Tyler JohnAneece, Itiya PLog, TorgrimAnfuso, GiorgioLogan, TimothyAngela, MertigLoh, WenyinAngelici, Maria CristinaLoh, VenursAngermann, JeffLohela-Karlsson, MalinAniello, FrancescoLöhmus, MareAnifandis, GeorgeLohwacharin, JenyukAnsa, BenjaminLoman, AbdullahAnsah, John PastorLombard, Melissa AAnsquer, Jean ClaudeLondono, BerlinAnthonj, CarmenLong, HaiyingAnticó, EnriquetaLongden, ThomasAntipova, AngelaLongo, EgmarAntonakis, GeorgiosLongstaffe, James G.Antoñanzas, FernandoLood, RolfAntonelli, AlessandroLopez, GabrielaAparicio García, Marta EveliaLopez Lopez, DanielApollonio, CiroLópez Sánchez, Guillermo F.Apostolopoulos, YorghosLópez-Cevallos, DanielApperson, CharlesLópez-Huertas, María RosaAppleton, KatherineLópez-López, DanielApter, AlanLopez-Quilez, AntonioArana, InesLopez-Rocha, SandraAranki, DanielLoppi, StefanoArce-Fernández, ConstantinoLopreite, MilenaArcher, DavidLops, FrancescoArchMiller, Althea A.Lorig, KateArcury, ThomasLorincová, SilviaArd, KerryLorini, ChiaraArdiles, PaolaLorz, CorinaArdito, LorenzoLoton, DanArghir, Oana CristinaLotto, RobynArias, Alexander GilLou, FangfeiArias-Pulido, HugoLouie, JessicaAriga, KatsuhikoLouis, JosephArion, FelixLouis, JulienAristovnik, AleksanderLoumakou, MariaArmah, FrederickLoupa, GlykeriaArmstrong, ThomasLourenço, CéliaArnaert, AntoniaLourida, IliannaArnarsson, ArsaellLous, JørgenArnetz, JudyLouwagie, GoedeleArora, AmitLovallo, William R.Arráez-Aybar, Luis-AlfonsoLove, PennyArrebola, Francisco JavierLoveland, KateArrey, Agnes EbotabeLoveless, Jane ClaryArriaga, SoniaLovelock, KirstenArribas Galarraga, SilviaLovrecic, MercedesArtacho-Cordón, FranciscoLowden, ArneArthur, Susan T.Lowe, Adrian J.Arthur, FrankLower, TonyArumugam, SurendranLöytönen, MarkkuAryal, NabinLozano-Guerrero, Antonio JoséAschenbrenner, SteffenLu, HongxuAschenbrenner, AndrewLu, JianAschonitis, VassilisLu, QibinAshiru-Oredope, DianeLu, LiAshish, PathakLu, HongzhouAshley, DavidLu, JunAshley-Martin, JillianLucía, GutiérrezAslanidis, TheodorosLucic, LukaAspray, ThomasLugg-Widger, FionaAssanelli, DeodatoLugo, Ricardo GregorioAssari, ShervinLugo-Candelas, ClaudiaAssink, MarkLuigi Invernizzi, PietroAstolfi, AriannaLuís, Ana TeresaASUMADU-SARKODIE, SAMUELLuis Ubago, JoseAtmann, OxanaLuiz, Olinda Do CarmoAtrash, Hani K.Lukomska-Szymanska, MonikaAttwood, SophieLumbreras, HortensiaAtwood, Charles W.Lund, SandraAu, DoreenLundén, JanneAuchincloss, AmyLuo, JunAuger, NathalieLuo, ShuaiAugustine, Swinburne A. J.Luo, XiaosanAumond, PierreLuo, PingpingAune, DagfinnLuo, WentaiAung, Meiji SoeLuongo, FabriziaAurora, RajeevLuongo, GiuseppeAustin, Erin EdwardLushington, KurtAvent, MinyonLusk, MaryAvino, PasqualeLusk, AnneAw, Tiong GimLuthra, PriyaAydede, Sema K.Luukkonen, TeroAyers, Britni L.Lv, HuixiongAyonrinde, OyekoyaLv, ShenghuaAyonrinde, Oyekoya T.Lvova, LarisaAysa-Lastra, MariaLy, Sun YungAzam, LailaLyall, KristenAzeez, AdeboyeLynam, MaryAziz, Abdul RashidLyons, Paul E.Azrad, MariaLystad, Reidar P.Azul, Anabela MarisaM. Childs, LaurenAzzone, GiovanniMa, WanglinBaalbaki, ZeinaMa, XiaofuBabcock, RebeccaMa, Zheng FeeiBacelar, EuniceMa, LeBacevičienė, MiglėMa, GuanshengBackes, ToddMA, CHANGXIBackstrand, Jeffrey R.Ma, PingBadau, AdelaMa, JieBadau, DanaMa, ZhenliangBaddam, Suman K. R.Ma, LiBader, DanielMa, YongfengBaer, JudithMaalouf, ChadiBaeza-Velasco, CarolinaMabhala, MzwandileBaghdadi, Ziad D.Mabhaudhi, TafadzwanasheBagley, ErikaMabry, J.ErinBagnati, RenzoMacDonald, DouglasBagordo, FrancescoMacdonald-Clarke, ClaireBahmani, SaharMacek, MarkBai, LibiaoMachado, YoanBai, RenrenMachado, CarolinaBaigorri, RobertoMachimbarrena, Juan M.Bailey, CatherineMachimbarrena, Juan ManuelBailey, KiraMacías Seda, JuanaBailey, KylieMaciaszek, JanuszBailie, RossMaciejczyk, PolinaBaillie, LesleyMaciejewski, GrzegorzBaird, NicholasMackay, SallyBajerska, JoannaMackay, LisaBajpai, RichaMackelprang, JessicaBakar, ShuvoMackenbach, Joreintje DingenaBaker, RichardMackowicz, JolantaBaker, KellyMacLeod, Catherine A.Baker, KatherineMacMillan, FreyaBaker, RichelleMacori, GuerrinoBakht, MartinMactaggart, IslayBąkowski, JarosławMadaleno, MaraBakre, Abhijeet A.Madau, Fabio A.Balanay, Jo Anne G.Madden, Richard CawleyBalastegui, Manso AndreuMaddineni, PrabhavathiBalázs, PèterMadeira De Carvalho, Luis ManuelBalbus, JohnMadsen, WendyBalcazar, Hector G.Madsen, Charlotte BernhardBalcerczyk, AnetaMadureira, HelenaBaldassarre, AntonioMady, Carlos Eduardo KeutenedjianBaldwin, Debora R.Maekawa, RyoBalietti, AncaMaes, MarliesBalietti, StefanoMagazzino, CosimoBaliraine, FrederickMagnavita, NicolaBallardini, MarcoMagori, KrisztianBallou, JordanMaguire, RomaBalsamo, MichelaMaguire-Jack, KatieBalwicki, ŁukaszMahmood, AtiyaBanaitis, AudriusMahmood, TaufiqueBanerjee, SudipMahmoud, Alaa El DinBanerjee, AniruddhaMailey, EmilyBanerjee, RumanMainka, AnnaBannigan, KatrinaMaisha, BuumaBansal, Geetha P.Majewska, MariaBanwell, HelenMajumdar, IndrajitBao, YiMakarska-Bialokoz, MagdalenaBaraibar-Diez, ElisaMakate, MarshallBaran, MarzenaMakino, ShintaroBarberis, AlessandroMakita, KoheiBarbet, AnthonyMakkonen, UllaBarbieri, JacopoMaksymiuk, GabrielaBarca, EmanueleMakubuya, TimothyBarcelo, JuanMalave-Llamas, KarloBarcham, RichardMalbergier, AndreBarilli, AmeliaMalecki, KristenBarja, SalesaMaleika, WojciechBarkoukis, VassilisMalek, ŽigaBarksdale Boyle, ElizabethMalesios, ChrisovalantisBarloková, DankaMaleszka, AlicjaBarmpounakis, Emmanouil N.Mali, MatildaBarnas, EdytaMalinowska-Cieślik, MartaBarnes, JoMałkowska-Szkutnik, AgnieszkaBarnes-Josiah, DeboraMallet, MarcBarnett, John H.Mallow, OleBarney, ChantelMalmqvist, EbbaBarney, DavidMaloberti, AlessandroBarnoya, JoaquinMalone, KerryBarocas, BrianaMaltezou, Helen C.Baron, KellyMalvezzi, MatteoBarone, LaviniaMamen, AsgeirBar-Or, DavidMamudu, Hadii M.Barragán-Escandón, AntonioManakil, JaneBarrena Martínez, JesusManamperi, NimanthaBarrett, Ashley K.Manandhar, RejinaBarrett, Emily S.Mancianti, FrancescaBarrett, RonaldMancini, FrancescoBarrington, DaniMancini, MarcoBarron, JennieMancl, Lloyd A.Barros, NillaMandala, AshokBarros-Dios, Juan MiguelMandic-Rajcevic, StefanBarroso, Cristina S.Maness, Sarah B.Barry, JonMangano, FrancescoBarsanti, MicheleMankin, Richard W.Barst, BenjaminMannetti, LuciaBartholomew, JohnManning, LouiseBartlem, KateManono, BonfaceBartlett, JohnManousakas, ManousosBartlett, SheridanMansfield, EdwardBartlett, MichelleMantwill, SarahBartoněk, DaliborMantzari, EleniBartzas, GeorgiosMantzaris, Alexander V.Barwicki, JanManuel, Fernández López-VizcaínoBar-Zeev, YaelManyes, LaraBarzyk, Timothy M.Manzo, CiroBasavarajappa, Balapal S.Manzoni, StefanoBasco, Leonardo K.Mao, LiangBascompta, MarcMao, DeqiangBashford, CarolMaple, MyfanwyBashkin, James K.Marano, NinaBaska, TiborMaraver, FranciscoBassareo, Pier PaoloMarcantonio, MatteoBastante-Ceca, Maria JoseMarcias, GabrieleBasti, LeilaMarcovici, GenoBasu, PratyushaMarcu, GabrielaBatchelor, PaulMarella, MajulaBathrellos, GeorgeMargalef Jornet, MariaBatista, Bruno LemosMargaritis, EfstathiosBatista Jr., Miguel L.Margaritis, LukasBattacone, GianniMari, LorenzoBattista, GabrieleMariani, StefanoBattista, RebeccaMariano, Norzagaray CamposBattle, KatherineMarín, Luz StellaBaudrot, VirgileMarinho, DanielBaudry, GinoMarini, MartinoBauer, StefanieMarion, Jason W.Baumgardt, JohannaMarken, FrankBausys, RomualdasMarkevych, IanaBaviera-Puig, AmparoMarkov, Marko S.Bayleyegn, TesfayeMarkowska-Szczupak, AgataBazire, AlexisMarotta, ClaudiaBeach, RobertMarouli, ChristinaBeale, LouisaMaroulis, JerryBeard, Renée LynnMaroyi, AlfredBeardsmore, CarolineMarques, NadejdaBeasley, ValMarques-Sanchez, PilarBeata, Kalska-SzostkoMarranzano, MarinaBeatty, P. RobertMarrin, Donn LouisBeauchamp, JonathanMarrs, CarlBecerra, MonideepaMars, MauriceBédard, AnnabelleMarston, Christopher G.Bednarczyk, Robert A.Marston, LouiseBeen, Jasper V.Marszelewski, WłodzimierzBegoray, DeborahMartelletti, PaoloBehnood, AliMartellotta, FrancescoBeil, KurtMartenies, Sheena E.Bejerot, SusanneMartienssen, MarionBekele, DawitMartin, RyanBekmuratova, SarbinazMartin, Richard H.Belak, AndrejMartin, WandaBelan, IngridMartin, DaveBelay, BrookMartinetti, AlbertoBelcher, John R.Martinez, PriscillaBeleña, María ÁngelesMartinez, Antonio J. TorijaBell, SimonMartínez Sanz, José MBell, Margaret RMartínez-Austria, Polioptro F.Bellavia, DanieleMartínez-Espinosa, Rosa MaríaBellettiere, JohnMartínez-Ferrer, BelénBelo, SilvanaMartínez-Graña, AntonioBełtowski, JerzyMartínez-Guitarte, José-luisBeltrán Alacreu, HectorMartinez-Hurtado, J.L.Bend, JohnMartínez-Ibarra, EmilioBenden, MarkMartínez-Lavín, ManuelBender, NicoleMartinez-Millana, AntonioBengel, JürgenMartínez-Nova, AlfonsoBenitez Arciniega, AlejandraMartinez-Triguero, JoaquinBenjamin, ElliotMartin-Gamboa, MarioBennet, Paul J.Martín-Hernández, DavidBennett, JulieMartinho, Vítor João Pereira DominguesBentel, Jacqueline M.Martin-Kerry, JacquelineBento, Douglas MayerMartin-Pena, AlfonsoBerardino, SantinoMartins, NatáliaBerchialla, PaolaMartinsson, JohanBerecki-Gisolf, JannekeMarvasi, MassimilianoBereitschaft, BradleyMarwarha, GurdeepBerens, Pamela D.Marzi, Isabel AlexandraBerens, Eva-MariaMas Mestre, JoanaBeretta-Piccoli, MatteoMasabni, JoeBerezuk, AndreMasago, YoshifumiBergamaschi, EnricoMasanotti, GiuseppeBergen, DorisMascherini, GabrieleBergeron, GlenMasciopinto, CostantinoBergés Tiznado, Magdalena ElizabethMasferrer, LauraBergstrom, CassendraMaslow, Joel N.Berke, OlafMasoero, MarcoBerkemeyer, ShomaMason, Howard J.Berkowsky, RonaldMason, LauraBerlin, Kathryn L.Masselli, ClaudiaBerman, JesseMasten, SusanBerman, TamarMasters, NicoleBernardi, GiulioMasterson, ErinBerninger, NathalieMasterson, DanielBernitsas, EvanthiaMastroianni, KarenBernotienė, EivaMasuhara, NaokiBernstein, Eve R.Mateo-Lázaro, JesúsBerntzen, LasseMateos, EsperanzaBerrett, Andrew N.Mateos, RaimundoBerry, ChristopherMateus, Dina M. R.Bertea, CinziaMather, CareyBerteotti, ChiaraMatheson, AnnaBerthelot, LaurelineMathews, Anne E.Bertoni, GiuseppeMathieson, StephanieBesenyi, GinaMathis, Mary WBessa, LucindaMathonnat, JackyBessa, FilipaMathur, CharuBest, CatherineMatinolli, Hanna-MariaBest, RussMatranga, DomenicaBettiol, Silvana S.Matsui, KenjiBeuhler, Michael C.Matsushima, AyamiBeutell, Nicholas J.Matt, MonikaBevilacqua, CarmelinaMattingly, Stephen PBewick, BridgetteMatyas, CoreneBex-Reimert, ViolaMatz, CarlynBeyea, JanMaucieri, CarmeloBeyerlein, AndreasMaund, KimBeylich, Achim A.Mauny, FredericBezerra, BarbaraMauree, DasaradenBezner, JanetMavaddat, NahalBhalla, NikhilMavridis, KonstantinosBharaj, PreetiMavrogonatou, EleniBhat, SushanthMawson, AnthonyBhatia, JatinderMay, ChristineBhatia, KulMaya-Jariego, IsidroBhave, DevayaniMaya-Treviño, María De LourdesBhuiyan, AzadMayer, GhislaineBiagi, FedericoMayer, AudreyBian, QijingMaynard, Olivia M.Bian, HuishengMayo, RachelBianco, CarloMazaheri, MandanaBianco, AntoninoMazilescu, Crisanta-AlinaBidarimath, MallikarjunMazur, JoannaBidwell, Amy J.Mazur, ArthurBiegel-Engler, AnnegretMazzei, RosalindaBielinis, ErnestMazzilli, FernandoBielory, LeonardMazzulla, GabriellaBień, AgnieszkaMbulo, LazarousBiernasiuk, AnnaMcAloney-Kocaman, KareenaBigelow, Ann E.McArdle, StephanieBigelow, PhilipMcAreavey, RuthBilal, EssaidMcAuley, DannyBilal, MuhammadMcBride, KateBilardi, Jade E.McCabe, BrianBilbao, JuliaMcCarthy, HelenBilliard, MichelMcCarthy, PumtiwittBilliot, ShanondoraMcCarty, ChristopherBimonte, GiovannaMcCarty, CatherineBindawas, Saad M.McCawley, MichaelBirch, BrianMcClintock, KahuBirenboim, AmitMcComb, JacalynBirkeland, EinarMcCrabb, SamBirkett, MelissaMcCrae, NiallBischoff, KarynMcCullagh, Marjorie C.Bishehsari, FarazMcCullough, BrianBishop, Jason C.Mcdonald, TedBiswas, BhabaMcDonough, Carrie ABiudes, Marcelo SacardiMcElhinney, EvelynBiziuk, MarekMcElroy, JaneBjørke Monsen, Anne-LiseMcElwee, KevinBlackall, PatrickMcgill, William B.Blackman, RossMcGinley, JaredBlacksell, StuartMcGrail, Matthew M.Bláha, LadislavMcIntosh, E. David G.Blake, DeniseMckay, FionaBlanchet, RosanneMcKay, CarlyBlanco, Juan A.McKeganey, NeilBlandin, GaetanMcKenna, Malachi J.Blanton, CynthiaMcKenzie, DianaBlinka, LukasMcKeown, MickBlok, David J.McLaren, MargaretBlood, WarwickMcLellan, DeborahBlukacz-Richards, E. AgnesMcLeod, GeriBlum, Jason L.McLeod, Geraldine F. H.Bluyssen, PhilomenaMcLeroy, KennethBoan, DavidMclver, KerryBoanca, PaunitaMcManus, BenjaminBoard, MaryMcManus, HamishBoardman, JedMeade, BenjaminBobinaite, ViktorijaMeads, Catherine A.Bockerman, Petri RikharMears, Stephen A.Böckmann, MelanieMedici, SerenellaBodar, CharlesMedina Quero, JavierBodas, MoranMedina-Vera, MyriamBoente, CarlosMehiriz, KaddourBogacki, JanMehlhorn, HeinzBogacki, MarekMehlig, KirstenBogdanovica, IlzeMeijer, ElineBogdański, PawełMei-Po, KwanBoggs, Douglas L.Meirhaeghe, AlineBogliolo, MassimoMekary, Rania A.Bohnet, IrisMelby, MelissaBohr, AdamMelchiorre, ErikBojar, Ana-VoicaMelendez, RitaBolboaca, Sorana D.Meliker, Jaymie R.Boldemann, CeciliaMellen-Vinagre, TeodorBolhuis, DieuwerkeMeloni, ItaloBollati, ValentinaMemon, TosifaBolton, JamesMenai, MehdiBonadonna, LuciaMendelsohn, Robert O.Bondoc, IonelMendes, JérômeBonetta, SaraMendes, Larissa LouresBongaerts, BrendaMéndez, Loyda B.Bonham, JimMéndez Mateo, InmaculadaBonham, JenniferMéndez-Lázaro, Pablo A.Boniface, SadieMendoza, DanielBonn, StephanieMendoza-Cano, OliverBonzini, MatteoMendy, VincentBooth, SueMeng, XiangchaoBorbély, YvesMennerich, ArturBorda, AnnMercado-Carmona, JesúsBoreham, Douglas R.Merkel, BroderBorena, Wegene T.Merklinger-Gruchala, AnnaBorg, JohanMerriman, TonyBorghi, FrancescaMerry, MelissaBorgmann, StefanMesbah, MortezaBorisenkov, Mikhail F.Meseguer-Santamaría, María-LeticiaBorisoff, JaimieMesquita, JoãoBorman, BarryMesquita, ConceiçãoBorn, KarenMessersmith, AmyBorn, PatriciaMessina, GabrieleBornioli, AnnaMessini, Christina I.Bornman, RianaMestres, ConxitaBoroneant, ConstantaMetin, BarisBoroń-Kaczmarska, AnnaMetz, Stefan W.Borrelli, LuigiMeyer, KevinBorrmann, ThomasMeyer, StephenBorschmann, RohanMeyer, Rosan WBortey-Sam, NestaMeyn, LeslieBortkiewicz, AlicjaMezzanotte, ValeriaBorzabadi-Farahani, AliMi, ZhifuBos, ElskeMi, HongBoscoe, FrancisMicek, AgnieszkaBossarte, RobertMichael, BryaneBossew, PeterMichael Hall, C.Bosso, LucianoMichalski, GrzegorzBostean, GeorgianaMichelangeli, AlessandraBotelho, Monica C.Michnik, JerzyBothe, DeniseMieczan, TomaszBotts, RyanMielgo-Ayudo, JuanBouaïcha, NoureddineMielgo-Ayuso, JuanBouamra, OmarMielke, Howard W.Boucaut, RoseMietlicki-Baase, ElizabethBouchard, DanielleMiettinen, Ilkka T.Bouchard, CatherineMignon, SylviaBouras, NickMiguel, SilviaBourgeois, DenisMijiritsky, EitanBourgoin, MickaelMikalef, PatrickBourliva, AnnaMilazzo, AdrianaBowatte, GayanMiles, DebraBowen, DeborahMiletta, MariaBowling, HeatherMilgrom, PeterBowman, Andrew S.Milham, PaulBoy, ErickMilhausen, RobinBoyas, Javier FranciscoMilici, Frances FlemingBoyaval, SébastienMilla, SaajanahoBoyd, HeatherMillar, GraemeBoziaris, IoannisMillar, J. CameronBozlaker, AyseMillecamps, MagaliBrabec, MarekMiller, JustinBracci, MassimoMiller, Anthony B.Braczkowski, RyszardMiller, KirkBradby, HannahMiller, CharlesBradley, ClareMiller, Kathleen E.Brådvik, LouiseMiller, BradleyBrady, OliverMiller, MarkBrahmbhatt, Tejal S.Mills, KathrynBraks, MarietaMilne, NikkiBrambilla, GiovanniMin, JungwonBrambilla, AriannaMinatoya, MachikoBramness, Jørgen GustavMinciullo, MarcoBranco, Manuel CasteloMingo, AntonioBrand, TilmanMinicozzi, PamelaBrandhagen, MartinMininno, ValeriaBrands, EdwinMinisola, SalvatoreBrangan, SanjaMiousse, IsabelleBranthwaite, HelenMir, DebbyBrantsaeter, Anne LiseMira, José JoaquínBrasington, DavidMiralles, Conxita MestresBratt, AlisonMirlohi, SusanBrattich, ErikaMirzoian, ArmenBråtveit, MagneMishra, AvinashBraun, JosephMisra, RanjitaBraun, MarkusMistiaen, WilhelmBraun-Lewensohn, OrnaMistri, AmitBraverman, EricMitchell, ConstanceBravo-Ureta, Boris E.Mitchell, CarolineBrazo-Sayavera, JavierMitra, RaktimBrčić Karačonji, IrenaMitroo, DhruvBregenzer, AnitaMitrou, FrancisBreinlinger-O’Reilly, JochenMitsakou, ChristinaBreitner, SusanneMlera, LuwanikaBrenna, MarcoMlocicki, DanielBrewer, GeorgeMoaddab, MahsaBriançon, SergeMobasheri, AminBridle-Fitzpatrick, SusanMóczár, CsabaBrimblecombe, JulieModenese, AlbertoBriner, WayneModhiran, NaphakBringolf, BettinaModica, GiuseppeBrinkerhoff, JoryMohanty, SaliniBrinn, MalcolmMohit, BabakBrinthaupt, Thomas M.Mohiuddin, MuhammadBrito, GraçaMöhner, MatthiasBroderick, PatriciaMoin, David AnssariBrodsky, BethMojiri, AminBroe, TonyMokkenstorm, JanBronzaft, ArlineMolenbroek, JohanBrooke, JoanneMolina, Jose LuisBrooks, PhilipMolina Garcia, JavierBrooks, KerryMolina-Mula, JesúsBroom, IainMoliner, CristinaBrough, LouiseMolino, MonicaBrouwer, SandraMöller, SörenBrown, MollyMøller, MetteBrown, MarkMollerup, SteenBrown, Kerry AnnMolnár, MónikaBrown, Scott C.Mölter, AnnaBrown, Mary JeanMomper, Sandra L.Brown, AndrewMonaghan, MaureenBrown, Candace S.Moncada, StefanoBrowning, MatthewMonda, VincenzoBrownstein, Catherine A.Mondal, SumonaBruce, AlisonMone, HélèneBruckner, Tim A.Monfoulet, Laurent-EmmanuelBrudzynski, KatrinaMoniruzzaman, MdBruland, DirkMonroy, FernandoBrumitt, JasonMonsees, ThomasBrunton, LisaMonsuez, Jean-JacquesBruserud, ØysteinMont, DanielBrzeska, JoannaMontag, ChristianBu, KaixuanMontcoudiol, NellyBucchignani, EdoardoMonteagudo, AndreaBuchanich, Jeanine M.Montes, AgustínBudhi, SridharMontevecchi, GiuseppeBugajski, PiotrMontuori, PaoloBugla-Płoskońska, GabrielaMonyeki, KotsediBührer, ChristophMoolla, RaeesaBui, AlexMoon, Kyong WhanBujnowska-Fedak, Maria MagdalenaMoon, Christopher J.Büke, TayfunMoore, RolandBuková, AlenaMoore, KristinBulkeley, KimMoore, Matthew D.Bullock, WilliamMoore, Brianna F.Bulltail, GraceMoore, JustinBundy, Joshua D.Moore, KarenBungum, TimothyMoore, PaulBunster, VictorMoores, CarlyBurge, SherwoodMorales Suárez-Varela, María M.Burgess, John A.Moran, MikaBurgette, JacquelineMoran, DanielBurghardt, KyleMorea, DonatoBurgherr, PeterMoreira, CarlaBurian, JaroslavMorel, BenoitBurkart, KatrinMoreno, Valentín MolinaBurkes, RobertMoreno-Andrés, JavierBurkey, Kent O.Moreno-Ruiz, DavidBurkhardt, SandraMorera, MariaBurkle, FrederickMoretto, AngeloBurnett, HarveyMorgado, Mariza G.Burnett, MeredithMorgan, Sara AfsharBurnley, StephenMorgan, RobertBurns, Ryan DonaldMorgan, MarshaBurris, MarkMorgan, Kerri A.Burstyn, IgorMorganti, MicheleBurszta-Adamiak, EwaMori, KazuhiroBurton, Elvin ThomaseoMorikawa, YukoBus, James S.Morisset, Anne-SophieBusby, ChrisMoro, SérgioBustamante, PacoMorote, ÁvaroBusu, MihailMorozov, IgorButler, RussMorphett, KylieButler, AinslieMorrell, StephenButler-Barnes, Sheretta T.Morris, Robert D.Butner, JennaMorris, MelanieButtiglieri, GianluigiMorris, CodyBye, RolfMorris, SheldonByers, Karin E.Morris, Brian J.Byne, William MMorris, David M.Byrne, SamuelMorris, georgeByrne, CatherineMorris, DavidByrne, RebeccaMorrissey, AnneCaballero, PabloMorrissey, KarynCaballero-Mora, María ÁngelesMorriss-Roberts, ChrisCabello, MariaMorrone, DomenicoCabo Verde, SandraMorse, Tracy D.Cabral, JaderMorse, DanielCabral-Pinto, Marina M. S.Moshammer, HannsCabrerizob, Francisco JavierMoskalik, TadeuszCacciottolo, MafaldaMosnier, AnneCaffarelli, CarlaMoss, SimonCaffery, LiamMoss, AntonyCahir, CaitrionaMossa, GiorgioCai, JialiangMossey, PeterCai, YutongMost, JasperCai, BaopingMostafa, SherifCai, YuzhuoMotta, Chiara MariaCaire-Juvera, GracielaMotyl, KatherineCalambokidis, JohnMou, HaiweiCalavalle, Anna-RitaMouhyi, JaafarCalder, RyanMourão, Paulo ReisCaldwell, Todd G.Mouraviev, NikolaiCalero-García, Francisco JavierMousa, AyaCallado, AndreMoutinho, VictorCallegari, CamillaMowe, MaxineCallejón, ÁngelMoyer, RachaelCallison, KevinMrhálek, TomášCalogiuri, GiovannaMrugalska, BeataCalvert, ThomasMudde, AartCalvi, RossellaMudunuru, Maruti KumarCalvo, AngelaMuehsam, DavidCalvo, ArleneMueller, NatalieCamacho Miñano, MaríaMueller, Megan P.Came, HeatherMueller, PieCamenga, Deepa RMugerauer, RobertCamenisch, Todd D.Muhajarine, NazeemCamera, DonnyMuhammad-Sukki, FirdausCamerino, DonatellaMuiesan, Maria LorenzaCameron, MichaelMukaratirwa, SamsonCameron, JoshMukherjea, ArnabCameselle, ClaudioMullan, JudyCamiciottoli, GiannaMullane, SarahCampa, FrancescoMüller, Jochen A.Campagna, CélineMüller-Alcazar, AnettCampagnaro, ThomasMulligan, AislingCampagnolo, DavideMullins, Raymond J.Campbell, Katarzyna AnnaMulyasari, FarahCampbell, MarilynMunblit, DanielCampbell, PaulMuncke, JaneCampbell-Arvai, VictoriaMundt, SabineCampisi, TizianaMuniz-Junqueira, Maria ImaculadaCamplain, RickyMuñoz, Cynthia E.Camporeale, RosaliaMuñoz-López, FranciscoCampos, Rui C.Munuera Martinez, PedroCampos, BrunoMurage, PeninahCanale, AntoninoMurante, Anna MariaCaneiras, CátiaMurata, ChiyoeCangkrama, MichaelMurbach, ManuelCanha, NunoMurff, SharonCannistraro, MauroMurgante, BeniaminoCanonico, EstherMurillo Tovar, Mario AlfonsoCantisani, GiuseppeMurmura, FedericaCanullo, LuigiMuro Jr, AldoCao, YangMurphy, KarenCao, JieMuscat, DanielleCao, YongsongMusher-Eizenman, DaraCao, LiqunMusicco, MassimoCao, QinguiMusk, BillCao, ChunxiangMusmarra, DinoCao, BolinMustapha, AdetounCaparros-Martin, JoséMuthaiyan, ArunachalamCapodaglio, AndreaMutie, MosesCapolupo, AlessandraMyers, SandraCappello, TizianaMyszkowska-Ryciak, JoannaCapra, Gian FrancoNabaweesi, RosemaryCapuano, EdoardoNaccarati, AlessioCaputo, JesseNadais, HelenaCarattini, StefanoNadhim, Evan A.Carboni, LuciaNadimpalli, Siva P.V.Carcillo, Joseph A.Nagaike, TakuoCarfora, ValentinaNagano, Marcelo SeidoCariñanos, PalomaNagata, KoseiCariou, StéphaneNair-Shalliker, VisaliniCarlin, DanielleNakai, KunihikoCarling, Gregory T.Nakai, SatoshiCarlos Alberola, MarNakajima, MasamiCarlsen, Hanne KrageNakamae, TakashiCarlson, Barbara E.Nakamura, MasashiCarmo, HelenaNakanishi, MiharuCarneiro, Sueli Coelho Da SilvaNalecz, HaniaCarnés, JerónimoNallasamy, PalanisamyCarnì, Domenico LucaNam, TaewooCaron, RosemaryNam, SangboCarotenuto, MarcoNandi, SaikatCarpenter, Elisabeth CounselmanNante, NicolaCarpentier, RodolpheNaponelli, ValeriaCarra, Maria ClotildeNaranjo Gómez, José ManuelCarrero, Jose AntonioNaranjo Hernández, José EugenioCarrillo-Castrillo, Jesús AntonioNardone, NatalieCarson, FraserNärvänen, ElinaCarswell, AndrewNash, Sarah H.Cartalis, ConstantinosNassan, FeibyCarter, William P.L.Natale, AldaCartwright, KimNatale, VincenzoCarugno, MicheleNaumann, ElkeCaruso, Joseph A.Naushad, MuCarvajal-Trujillo, ElenaNavab-Daneshmand, TalaCasanovas, CarmenNavalta, Carryl P.Casas, IreneNavalta, JamesCasati, LaviniaNavarrete-Muñoz, Eva MªCascone, StefanoNavarro-Pérez, Carmen F.Case, BruceNavi, MaryamCaserta, DonatellaNawaz, MianCassidy, TonyNazeer, MajidCassidy, SophieNebeská, DianaCastellano, OrlandoNędzarek, ArkadiuszCastellano, Javier GarcíaNeelameggham, Neale RCastelli, GiulioNegishi, TakayukiCasteren, Jan VanNeiva, Henrique PereiraCastillo-Salgado, CarlosNejezchlebová, HelenaCastro, Shamyr SulyvanNemeth, LynneCastro Sánchez, ManuelNémeth, LászlóCatania, GianlucaNersesyan, Armen K.Catherine, HayesNesticò, AntonioCatley, DelwynNeubauer, AndreasCauli, OmarNeumann, NormanCausby, RyanNeunert, GrazynaCava, María J.Neves, Antônio AugustoCavaleiro Rufo, JoãoNewins, AmieCavallaro, FaustoNewman, John F.Cavicchi, CaterinaNewman, GalenCavoretto, PaoloNewman, AndrewCayón De Las Cuevas, JoaquínNewson, LisaCeballos, DianaNg, KwokCebula, JanNg, BetsyCejudo, JavierNg, Lisa C.Celeste, Roger KellerNg, Isabella F. S.Cella, Silvano GabrieleNgo, Anh PCentobelli, PieraNgueta, GerardCentofanti, Stephanie A.Nguipdop-Djomo, PatrickCeptureanu, Sebastian IonNguyen, Vu H.Ceranowicz, PiotrNguyen, Jenn L.Cerchione, RobertoNguyen, Dang-MaoCerda, EmilioNi, DebingCerdà, ArtemiNí Shé, ÉidínCerel, JulieNiazi, NabeelCeresia, FrancescoNicholas, AngelaCerna, MiloslavaNicholls, KeithCerniglia, LucaNichols, Michelle G.Cerri, CesareNichols, DavidCervera Juanes, Rita PNichols, JenniferCervero Aragó, SílviaNicolau, AnaChadborn, NeilNicolini, AndreaChadda, NeeruNie, PengChadwick, PhilipNie, JinleiChae, HeeyoungNieboer, EvertChafe, Zoe AnnaNiedermeier, MartinChai, RifaiNiedzielski, PrzemysławChakraborty, RajaNielsen, MichaelChalah, MoussaNiemiec, ChristopherChamberlain, JimNieminen, PenttiChambers, MaryNienhaus, AlbertChan, Chien-LungNieto, GemaChan, King MingNieves, KourtneyChan, IsabelleNighbor, Tyler D.Chan, Alan H. S.Nijmeijer, ArianChan, Siew-PangNikolaou, IoannisChan, Heng Choon (Oliver)Nilsson, AndreasChander, HarishNishi, MayumiChandran, ArunaNishino, TatsuyaChang, KaowenNobmann, Elizabeth D.Chang, Po-JuNoel, AlexandraChang, Hsiao-yun AnnieNogawa, KazuhiroChang, Lisa Tzu-ChiNoh, SoowoongChang, Geng-RueiNolan, ClodaghChang, AngelaNomura, KyokoChang, Wen-DienNones, MichaelChang, Ta-YuanNooijen, CarlaChang, Chien-ChungNoonan, RobertChang, Hui-huaNoori, NavidehChang, Chih-chenNorbäck, DanChang, Shu-ManNorful, Allison A.Chang, Chih-ChingNorihiko, NaritaChang, Fu-KueiNorman, TrudyChang, Jer-MingNorman, TrevorChao, Tzu-YuanNorris, EmmaChao, How-RanNosetti, LuanaChao, JianqianNosi, CostanzaChao, Li-ChangNøst, Therese HaugdahlChappell, GraceNøstbakken, Ole JakobCharalampopoulos, IoannisNotaro, SandraCharles, Luenda E.Notley, Caitlin JadeCharlotte, WunderinkNotredame, Charles-EdouardCharmas, MalgorzataNourazari, SaraCharvat, Robert A.Nouri, Andre SantosChasin, MarshallNovak, PriscillaChater, PeterNovío, SilviaChatterjee, SatabdiNovo, Luís António BalreiraChatterjee, AvikNový, Ing. PavelChau, Kwok-wingNowak, GlenChaudhuri, NaziaNowak, Felicia V.Chauhan, SmitNowalk, Mary PatriciaChauhan, MunishNowicka, GrażynaChaves, Luis FernandoNowossadeck, EnnoChe, WeitaoNowotarski, ShannonChen, JiangangNthunya, Lebea N.Chen, KaiNtila, SithandiweChen, XiangNtougias, SpyridonChen, ZuosongNübling, RüdigerChen, LingxinNucci, DanieleChen, Kow-TongNüesch-Inderbinen, MagdalenaChen, ChiachungNugnes, FrancescoChen, ChunNugraha, AdiChen, DingjiangNuić, IvonaChen, CongNuñez, NeftaliChen, Kuang-ChiNúñez, Remigio ParadeloChen, Nai-HuaNúñez Pérez, José CarlosChen, LeiNúñez-Delgado, AvelinoChen, LongNurulnadia, Mohd YusoffChen, Tung-tsanNuutila, JaakkoChen, YaoningNuytemans, KarenChen, Yi-TuiNwanodi, OromaChen, YanNygaard, BirteChen, WenchunO´Higgins, JamesChen, Tzeng-JiO’Konek, Jessica J.Chen, Yen-FuO’Mahony, MicheálChen, FengOancea, S. CristinaChen, Xiang "Peter"Obeng, Aniwaa OwusuChen, RuishanObeng-Gyasi, EmmanuelChen, BoOberle, Crystal D.Chen, Ching-HsienObermüller, NicholasChen, QiO’Brien, KimberlyChen, Jung-WeiO’Brien, Katie M.Chen, MaosiObrycki, JohnChen, Jo - HuiOcampo, DiegoChen, ShuaiOchiai, HirokoChen, WeiO’Connor, MeredithChen, FeiO’Connor, JohnnyChen, Yu-ChunOctaria, RanyChen, ZhanghuaOda, EijiChen, Yi-ChunOdland, Jon ØyvindChen, WeihongO’Dwyer, JeanChenal, JérômeOe, MisariCheng, LiangO’fallon, Liam R.Cheng, DelfineOgeil, RowanCheng, TianhaiOgino, ShujiCheng, HelenOgneva-Himmelberger, YelenaCheng, I-ShiungÖgren, MikaelChengwen, SunOgtrop, FlorisCheong, Yuen KiOguma, YukoCheong, Hae-KwanOgunrinde, JoyceChereddy, Kiran KumarO’Hagan, JohnCherniack, Evan PaulO’Hare, AnthonyChernyak, SergeiOhba, RyohjiCherrie, JohnOhnishi, MasatoshiCherukupalli, RajeevOjeda-Benitez, SaraChetwynd, AndrewOkajima, HideakiCheung, KinOkrasa, MałgorzataCheung, Ting On LewisOkuda, MasayukiCheung, LiOkunade, Albert ACheung, Lewis T.O.Olejniczak, DominikChhikara, SudeshOleniacz, RobertChi, Kai-HsienOlišarová, VěraChiang, ChifaOlivas, Raul NavarroChiang, Li-ChiOliveira, Paula Alexandra Martins DeChiang, Hung-LungOliveira, José António VasconcelosChiang, Chien-HsiehOliveira, Ana BeatrizChiarini, AndreaOliver, BrianChica-Olmo, JorgeOliveto, GiuseppeChiefari, EusebioOlmedilla Fernández, MaríaChien, Chun-RuOlsen, Sonja J.Chien, Yi-wenOlsen, JonathanChild, StephanieOlson, Michael W.Chileshe, NicholasOlu, OlushayoChilibeck, PhilOluwole, OluwafemiChillón, PalmaOmagari, KatsuhisaChim, Hui QingO’Malley, LucyChinniah, YuvinOmboni, StefanoChipps, JenniferOmodior, OghenekaroChiu, Marcus Yu LungOmoruyi, FelixChiu, Weihsueh A.Omotayo, MoshoodChmielowski, KrzysztofO’Mullan, GregoryCho, Do YeonOncioiu, IONICACho, ClareO’neill, EoinCho, YoungOngel, AybikeCho, Gi-HyougOno, Mario AugustoChoate, Peter W.Onozuka, DaisukeChochlakis, DimosthenisOon, Hazel H.Chohan, Balwant S.Oosthuizen, JacquesChoi, Seung UkOpdyke, AaronChoi, Jeong DongOpoku, AlexChoi, KelvinOpoku-Duah, StephenChoi, Nag JungOppliger, AnneChoi, SungOpree, SuzannaChoi, Yun-JungO’Reilly, RebeccaChoi, KwangyulOren, EyalChokkanathan, SrinivasanOri, GudesChong, Ngee SingOrmsbee, FloydChong, Marc Ka-chunOrnelas, YoshiraChoo, CarolOropeza-Perez, IvanChoo, Pei LingOrton, ElizabethChopra, Shauhrat S.Orton, SophieChoudhary, Kumari SonalOrtuño-Sierra, JavierChow, AngelaOrupõld, KajaChrisinger, Benjamin W.Orzolek, Michael D.Christiansen, HannaOsborne, NicholasChristoph, MaryOsca, AMPAROChruściel, PawełOsganian, Stavroula K.Chrzanowski, ŁukaszOshiki, MamoruChu, Chun-HungOsier, NicoleChua, Tze-ErnOsma, JorgeChuang, Kai-JenOsmani, Ahmad ReshadChuang, Ting-WuOsmond, PaulChuang, Li-MinOsório, HugoChuang, Shu-ChunOsornio-Vargas, Alvaro R.Chuang, Hung-YiOssiannilsson, EbbaChui, KevinOstberg, JohanChung, YounshikOSullivan, LeonardChung, Hsin-FangOteng-Peprah, MichaelChung, Yat-NorkOtsuki, TakemiChung, Yang WoonOu, Yih-ChangChurch, E. MitchellOubiña, JavierCiaccio, MarcelloOuyang, XiaoguangCianchetti, CarloOvalle, María AntoniaCiccarese, GiuliaOverholser, James C.Ciccone, Marco MatteoOverholser, JamesCicero, Arrigo Francesco GiuseppeOviedo, OscarCiciliot, StefanoOwili, PatrickCiciurkaite, GabrieleOzaki, NoriatsuCiesielczuk, TomaszOzturk, YagmurCieszewska, AgataPaavilainen, EijaCimiotti, Jeannie P.Pace, NelsonCimochowicz-Rybicka, MalgorzataPacheco, Fernando A.L.Cinelli, PatriziaPacurari, MaricicaCinnamon, JonathanPaczkowski, SebastianCiocan, CorinaPadula, AmyCipollina, MariaPafka, ElekCirés, SamuelPaganelli, FilippoCiriaco, FulvioPagano, IanCisterna, PedroPagano, FrancescaClaassen, LiesbethPage, KristenClaiborn, CandisPagliarin, SofiaClapham, Phillip J.Palamar, Joseph J.Clark, CainPalazoglu, AhmetClark, Nicholas J.Palazzolo, Dominic L.Clark, Cain C TPalidda, SalvatoreClarke, NaomiPalma, CarlaClarke, NeilPalma, Luca DiClaro, RafaelPalma, AlexandreClaros, EdithPalmen, NicoleClauß, MarcusPalmer, MelissaClay, Lauren A.Palmieri, MarcoClayton, SusanPalomo, LeenaCleary, Paul D.PALOMO LOPEZ, PATRICIAClemens, RogerPamucar, DraganClimie, RachelPan, MeixiaCluff, LauriePan, XiCoban, OksanaPan, HaozhiCobb, Julie Turner-Pan, Wei-PingCobelens, FrankPan, YongchuCocco, PierluigiPan, Wen-ChiCoccolo, SilviaPan, ShuaiCoelho, Denis A.Panagopoulos, Dimitris J.Coelho, Ana CláudiaPanagopoulos, ThomasCoggiola, NadiaPanagos, PanosCohen, Harold C.Pandey, Krishan K.Cohen, LauriePandit, ArkaCohen, DavidPani, Shantanu KumarCohen, Arnon D.Paniagua, Jorge OchoaCol, Nananda F.Panis Panis, LucColavita, GiampaoloPankratz, CurtisColder Carras, MichellePannek, JürgenCole, RoyPant, Puspa RajCole, RachelPantelopoulos, AthanasiosCole-Hunter, TomPanza, FrancescoColeman, DavidPaolini, RiccardoCollado-Yurrita, LuisPapadakaki, MariaCollings, PaulPapadaki, AngelikiCollins, LaPorchiaPapadakis, StamatisCollins, SamuelPapadakis, RaffaelloColodro-Conde, LucíaPapadopoulos, AndrewColombani, NicoloPapadopoulos, NikosColombo, MarcoPapantoniou, PanagiotisColomer, Francisco JosePapapostolou, VasileiosColson, NataliePaparusso, AngelaColucci, ErminiaPapassotiriou, Ioannis G.Columna, LuisPappas, Richard StevenColvin, AliciaPapurello, DavideComan, EmilParajuli, RajendraComfort, AlisonParajuli, DurgaComstock, SarahParajuli, SandeshConceição, Eusébio Z.E.Paramithiotis, SpirosConnell, James E.Paraskevopoulou, DespinaConrad, ZachParazzini, MartaConroy, SimonParent, André AnneContardo Ayala, Ana MaríaParise, Carol A.Contassot, EmmanuelParisi, AlfioConte, MariannaPark, SubinConti, ValeriaPark, SookukConti, SaraPark, BoyoungConti Gea, OliveriPark, Sue K.Contini, DanielePark, Jae BumConvertino, MatteoPark, Sin-AeCook, Amanda C.Park, SojungCook, MargaretPark, DaeryongCooper, Matthew J.Park, Young JooCooper, KayPark, Chan-SikCooper, David MartinPark, Youn ShikCope, MichaelParker, Elizabeth A.Coppi, AntonellaParker, Megan E.Corazon, Sus SolaParker, JackCorazza, Maria VittoriaParks, ChristineCordero, JoseParlesak, AlexandrCordone, SusannaParmenter, BelindaCori, JenniferParr, Jeffrey J.Cori, LilianaParr, EvelynCork, SusanParry, SharonCorrea, Patricia Isabel MatusParsa, MahdiCorrea Marques, RejaneParvez, ShahidCorrea-Burrows, PaulinaPasanen, TyttiCorreia, Pedro Miguel Alves RibeiroPaschalidou, Anastasia K.Cortés Castell, ErnestoPaschalis, VassilisCortese, SamuelePascual Aguilar, Juan AntonioCorti, PaoloPaseiro-Cerrato, RafaelCortini, MichelaPasha, Evan P.Cosentino, CarloPashley, Richard MarkCosgrave, CatherinePasman, Hans J.Cosma, AlinaPassannante, MarianCosma, PinalysaPasti, LuisaCosta, Nuno Manuel Sessarego Marques DaPastor Vicedo, Juan CarlosCostabile, PierfrancoPastore, LuisaCosta-Black, Kátia MacielPatchay, SandhiranCostantino, ClaudioPatel, RiyaCostanzo, SimonPatel, MinalCostella, Marcelo FabianoPatel, DevenCoughenour, CourtneyPateraki, StylianiCoull, BrucePathak, Ram D.Coulter, JohnPati, DipanwitaCourtney, RonanPaton, DouglasCousins, RosannaPatrascu, VasileCoussens, ScottPatriarca, RiccardoCoutte, LoicPatro-Małysza, JolantaCowie, JuliePatsios, Sotiris I.Cowles, RobertPatton, AllisonCox, DanPatton, Allison P.Cox, SharonPaudel, ShishirCrabbe, HelenPaul, Narayan ChandraCraig, BrettPaul, BimolCramer, RobertPaul, Christopher JohnCraveiro, IsabelPaul, DavidCravero, Maria CarlaPaulesu, LuanaCremasco, Margherita MichelettiPavagadhi, ShrutiCrisafulli, ConcettaPavón García, IgnacioCrisp, Dimity A.Pawar, KasturiCriss, ShaniecePawlaczyk-Łuszczyńska, MałgorzataCrocombe, LeonardPayne, JenniferCroft, BevinPazdro, KseniaCromar, KevinPazzaglia, FrancescaCronin, AidanPeacock, GeorginaCronkite, Ruth C.Pearce, Dora ClaireCrooks, GeorgePearson, NatalieCross, AdamPeck, Richard M.Crossan, ClairePeckham, StephenCrouch, AlanPeckham-Gregory, Erin C.Crowe, JessicaPecora, Peter J.Crozier, Alyson J.Pedisic, ZeljkoCruz-Topete, DianaPeel, JenniferCuberos, Ramón ChacónPehlken, AlexandraCucciniello, RaffaelePei, JiantaoCueto, José LuisPeide, LiuCueva, AlejandroPelecanos, LoizosCui, ZhibinPelfrêne, AurélieCui, CanPelikan, Jürgen M.Cui, YuxiaPellegrini, MarcoCukrov, NevenPeller, JulieCuller, CorinnaPellicer, EugenioCullerton, KatherinePeña García, AntonioCulpan, IanPeña Quintana, LuisCummings, ChristopherPeña-Martínez, RamónCummins, IanPence, BrandtCunha Rudge, Marilza VieiraPeng, ZheCunningham, CaitrionaPeng, JianCunningham, NatoshiaPeng, HongCuratolo, MichelePeng, LiCurcio, GiuseppePeng, ZeyuCurry, ScottPenkala, StefaniaCurtis, DenicePentakota, Sri RamCurtis, Chadwick C.Pentaris, PanagiotisCutrona, CarolynPepe, JessicaCvorovic, JelenaPepi, MilvaCybulski, MateuszPepper, Jessica K.Cycoń, MariuszPereira, AlcidesCyrus, ElenaPereira, LianeCzapik, PrzemysławPereira, Amaro OlimpioCzarnigowska, AgataPereira-da-Silva, LuisCzechowski, TomaszPereira-da-Silva, LuísD’Amato, AlessioPerez, BrittanyD’Amico, MarioPérez, PatricioD’Auria, Francesco.Pérez González, María EugeniaDa Cunha, Diogo ThimoteoPerez-Atayde, Antonio R.Da Lomba, SylviePérez-Belmonte, Luis M.Da Silva, Lucia Regina CangussuPérez-Fuentes, María Del CarmenDa Silva Guerra, Danielle ReginaPérez-Gil, JesúsDąbrowska, JolantaPerez-Gracia, Maria-TeresaDąbrowski, WojciechPerez-Heydrich, CarolinaD’Acci, LucaPérez-Sánchez, JulioDagdeviren, CananPerez-Tejero, JavierDaley, PeterPergadia, MicheleDalli, MaríaPerin, GiorgioDallman, AnnPeriyasamy, PalsamyDalton, Alice M.Perkins, WendyDaly, AlisonPerlman, DanDaly, Barbara MabelPerna, SimoneDamasio, EdilsonPernigotto, GiovanniDamiani, PaolaPero, RaffaelaDamma, DevaiahPerotto-Baldivieso, Humberto L.D’Andreagiovanni, FabioPerri, MarkDanek, TomasPerry, Simona L.Dang, ShaonongPersoskie, AlexanderDang, ZhiPertoldi, CinoDanielache, SebastianPerucka, IrenaDaniele, BrombalPescara-Kovach, LisaDaniele, AuroraPesola, FrancescaDaniels, TomPestana, DiogoDanilovich, Margaret K.Petach, HelenDanks, MarcellaPeters, ShawnDarbra, R.M.Peters, PatrickDarby, KatePetersen, AnneDarchia, NatoPetersen-Perlman, JacobDarling, JosPeterson, DerickDarlington, PeterPeterson, DanielDarmon, NicolePeterson, Emma LeeDaskalopoulou, IrenePeto, TundeD’Atri, AuroraPetousis-Harris, Helen AspasiaDaud, SuzannaPetrie, Joshua G.Daus, CatherinePetroselli, AndreaDavid, Annette M.Pettersson, DavidDavies, Anita A.Peyer, KarissaDavies, EmmaPezzuolo, AndreaDavies, MichaelPfeffer, KarenDavis, NoraPhalen, DavidDavis, NickPhibbs, Suzanne R.Davison, KarenPhilliber, SusanDawson, JohnPhinyomark, AngkoonDay, JohnPiantino Ferreira, Antonio JoséDe Almeida, Claudia RibeiroPiasecki, JessicaDe Andrade, MarizaPiccoli, GiorginaDe Berardis, DomenicoPiccolroaz, SebastianoDe Beurs, DerekPickett, DrewDe Castro, Nathália Santos SerrãoPieczka, AdamDe Castro, AlexandrePielech, RemigiuszDe Cocker, KatrienPiepenbrink, KurtDe’ Donato, FrancescaPietri, SylviaDe França, Elvis JoacirPietrobelli, AngeloDe Giglio, OsvaldaPietrucha-Urbanik, KatarzynaDe Gruijl, FrankPigeon, PatrickDe Jong, Lex D.Pigłowski, Marcinde Jonge, J. (Jan)Pignitter, MarcDe Kluizenaar, YvonnePikethly, AmandaDe Kort, Laetitia M.O.Pikoulis, EmmanouilDe La Fuente, AlbertoPillai, Vijayan K.De La Fuente, RenéPillay, ManikamDe La Rosa Carrillo, DavidPillow, David R.De La Vega, RocioPilz, StefanDe Loof, HansPimentel, GustavoDe Lourdes Gomes Pereira, MariaPinheiro, PauloDe Morais, Marcos Vinicius BuenoPinior, BeateDe Müllenheim, Pierre-YvesPinto, Josephde Nazelle, AudreyPinto, Paula C.De Oliveira, João Ricardo MendesPiotr, BugajskiDe Oliveira Fernandes, EduardoPiqueras Rodríguez, José AntonioDe Palma, GiuseppePirlott, AngelaDe Paola, PierfrancescoPirogova, ElenaDe Pascale, FrancescoPirola, LucianoDe Paula, Fabiana MartinsPirozzi, FeliceDe Peretti, ChristianPistelli, FrancescoDe Rosis, SabinaPitt, JohnDe Schepper, JeanPitteri, MarcoDe Toro, PasqualePitto, AndreaDe Valck, JeremyPiu, SahaDe Vuyst, StijnPivonello, RosarioDe Waard, VivianPizarro, AndreiaDe Wilde, KatrienPłachcińska, AnnaDean, Julie H.Platania, MarcoDe-Bandt, Jean-PascalPlaton, TiniosDeckers, StijnPlatt, StephenDeFelice, Nicholas B.Plaza-Díaz, JulioDefrancesco, EdiPlevoets, BieDeGuzman, Pamela BakerPliakas, Fotios-KonstantinosDeilami, KavehPlummer, VirginiaDeisenhammer, Eberhard A.Pluut, HelenDekker, MarloesPoetsch, AnsgarDekoninck, LucPoland, BlakeDeksissa, TolessaPolanska, KingaDel Gallo, MddalenaPolen, TerryDel Giudice, RitaPolesello, StefanoDel Hoyo, Carmen M.Polimeni, John M.Del Razo Jiménez, Luz MariaPolkinghorne, Benjamin G.Del Re, BrunellaPollard, K. MichaelDel Río Celestino, MercedesPollitt, KrystalDel Río Rama, María De La CruzPollmann, KatrinDelany, ClarePolo, GinaDelellis, NailyaPompili, MaurizioDelgado, JordiPonto, MariaDelgado-Angulo, ElsaPoole, Jill A.Deligeoroglou, EfthimiosPopeski, NaomiDeLisi, MattPorrovecchio, AlessandroDelmelle, EricPorsius, Jarry T.DeLong, GaylePortegijs, ErjaDelorme, VincentPortengen, LützenDelucia, GiuseppePortnov, Boris A.Deluka, AleksandraPosada-Quintero, HugoDemetrovics, ZsoltPosthuma, LeoDemiroglu, CenkPoteyeva, MargaritaDemková, LenkaPotter, JuliaDemlie, Molla B.Pottie, KevinDempsey, ChristopherPoulain, TanjaDendup, TashiPourret, OlivierDeng, JayPowell, HelenDeng, WuPowell, FayeDeng, HepuPowell, JaneDennington, SimonPowell-Wiley, Tiffany M.Denny, ClarkPowers, James S.Dentinho, TomazPowles, JohnDeo, Randhir P.Powrózek, TomaszDepner, Christopher MichaelPrabhu, AntonyDerevensky, JeffreyPradhan, RinaDerlein, AndreaPradhanang, SoniDeruyter, GretaPrasad, BablyDesai, AbhishekPrasse, CarstenDesapriya, EdiriweeraPrecioso, JoseDeschasaux, MelaniePressley, Joyce C.DeSesso, John M.Presto, AlbertDeShon, HeatherPrimeaux, Stefany D.Deshpande, MaithiliPrince, Roger C.DeSisto, Stephanie L.Prince, LatrinaDesrosiers, MelaniePrince, BennieDettori, MarcoPrinsi, BhaktiDettweiler, UlrichPrior, SarahDetzel, PatrickProbst, Tahira M.Deumlich, DetlefProchaska, CharikleiaDevaraj, SrikantProchaska, John D.Devaraj, AishwaryaProcter, PaulaDe-Ville, SimonProcter, Sandra B.DeWeese, RobinProcter-Gray, ElizabethDewhurst, SusanProdinger, Peter M.DeWitt, Jamie C.Protudjer, JenniferDey, AditiProyer, RenéDeYoung, SarahPrunuske, AmyDeziel, NicolePryss, RüdigerDhariwala, MiqdadPsaroulaki, AnnaDhillon, HaryanaPu, ZhengningDhiman, SaurabhPu, XujinDhital, NarayanPuciato, DanielDhuffar, ManpreetPuckett, Jae A.Di Agostino, SilviaPuddu, Paolo EmilioDi Bartolomeo, AntonioPuertas Molero, PilarDi Carlo, PaolaPugliese, FrancescoDi Corrado, DonatellaPulakka, AnnaDi Credico, BarbaraPulster, ErinDi Gennaro, FrancescoPuma, JiniDi Giacomo, DinaPurchas, Roger W.Di Giuseppe, DarioPurdy, MikeDi Luca, MarcoPuri, KritiDi Martino, FerdinandoPuzzolo, ElisaDi Nisio, AndreaPyrgaki, KonstantinaDi Pietro, AngelaQi, YanDi Sarli, Valeriaqian, wuyongDiaconu, KarinQiang, ZheDiakiese, Bobette MatulongaQiao, XianliangDias, Claudia CamilaQiu, GuangleiDías Pereira, LuisaQiu, JianhuiDiaz, MarvinQiu, JianyingDiaz, AnjoliiQiu, YongheDíaz, JulioQu, ShaojianDiaz-Sarachaga, Jose ManuelQuader, Mohammad AbdulDibai-Filho, Almir VieiraQuaranta, GiovanniDickerson, AishaQuartu, MarinaDickinson, KacieQueirós, AlexandraDiderichsen, FinnQuémerais, BernadetteDieberg, GudrunQuick, MatthewDieris-Hirche, JanQuijano, LauraDiessner, RhettQuince, Thelma A.Díez, Isabel De La TorreQuinlan, Jennifer J.Díez Escudero, JuliaQuinn, Margaret M.Dijkstra, AtzeQuinn, AshlinnDillon, DeniseQuiroz, DianaDilonardo, ElenaQuock, RyanDimitrios, VagenasRa, ChangsixDimitriou, EliasRabinowitz, SamuelDimopoulos, AndreasRabionet, Silvia E.Ding, YichuanRabkin, Judith GodwinDinis, Maria Alzira PimentaRace, MarcoDiniz, Márcio AugustoRacine, ElizabethDinkel, DanaeRacionero-Plaza, SandraDipnall, JoannaRada, Elena CristinaDirk, RichterRada, ElenaDixon, GradyRadcliff, Fiona JaneDluholucký, SvetozárRadford, Samantha A.Do, ThuyRadford, KylieDodd, RachaelRadhakrishnan, MohanasundarDoekes, GertRadicic, DraganaDoerr, CelesteRadulescu, CristianaDohrn, Ing-MariRadziemska, MajaDolcini, Giorgio AndreaRafiq, ShamailaDomingues, SaraRage, EstelleDomingues, PedroRaghunandan, MayaDonadio, CarloRaheem, BamideleDonat Vargas, CarolinaRahikkala, AnttiDonchin, MilkaRahman, MunsurDong, ShengkunRahman, RahbelDong, YiboRahman, Muhammad AzizDong, ZengchuanRahman, Ismail M.M.Dong, WeizhenRahman, Muhammad TauhidurDongen, RobertRai, VikrantDonin, AngelaRaiff, BethanyDonny, Eric C.Raina, SatishDonohue, MauraRaitasalo, KirsimarjaDonovan, RobertRajaraman, SivaramakrishnanDonzelli, GabrieleRajesh Lenin, RajiDoolan, MatthewRajurkar, MihirDoosti, HassanRakshit, SudiptaDopart, PamelaRalf, RisserDoran, NealRalli, MassimoDore, BettyRallo, GiovanniDorea, Caetano C.Ralston, Margaret L.Dórea, JoséRalston, Nicholas V. C.Dórea, FernandaRam, YaelDorling, DannyRamadas, AmuthaDorneich, Michael C.Ramakrishnan Geethakumari, PraveenDoshi, SaumilRamamurthy, PrathapDosoky, NouraRamanathan, SubhaDoucoure, SouleymaneRamirez, BernardoDoughan, BasselRamirez, MarizenDouglas, OwenRamírez, M. IsabelDouglas, PippaRamirez-Andreotta, MonicaDouwe Flapper, SimmeRamírez-Santana, MurielDoveri, MarcoRamírez-Vélez, RobinsonDowell, TonyRamkumar, ArputhamDowling, SallyRamoelo, AbelDozier, TomRamon, ShulamitDr. Grant, WilliamRamos, João António EstevesDragano, NicoRamos, VictoriaDragovich, DeirdreRamos, NiloDrapeau, Christopher WRamos, PedroDraper, JohnRamos Miras, Jose JoaquinDrapper, DarrenRamos Vidal, IgnacioDreassi, EmanuelaRamsay, Michael A.Dreibelbis, RobertRamsden, VivianDrevet, JoëlRamsey, ElizabethDrews-Botsch, CarolynRamseyer Winter, VirginiaDriesche, Sander Van DenRamtekkar, UjjwalDriezen, PeteRana, DipakDrigas, Athanasios S.Ranavolo, AlbertoDriver, KaraRanderson, PeterDrobnic, FranchekRandler, ChristophDrosdzol-Cop, AgnieszkaRanka, RenateDroste, NicRanmuthugala, GeethaDroste, NilsRanzato, EliaDrouiche, NadjibRao, SowmyaDrozdovitch, VladimirRao, AjithDryer, RachelRapisarda, VenerandoDu, HuRaposo, MariaDu, XinzhongRappazzo, KristenDuan, WeiliRäsänen, KimmoDuarte, AidaRashedi, EhsanDube, TimothyRashid, NaimDubey, RameshwarRashid, MebDubey, HarishchandraRasing, SanneDulebenets, Maxim A.Rask, Kimberly J.Duncan, MikeRasool, Sama FaizDunea, DanielRasul, AzadDunn, KateRathi, NehaDuplaga, MariuszRattray, BenDuquette, PierreRavaioli, StefanoDurand-Zaleski, IsabelleRaven, MelissaDuran-Valle, Carlos JavierRavuri, SudheerDurkin, Anne E.Rawla, PrashanthDurmus, DorukalpRay, MadelynDuron, JacquelynnRayson, Gary D.Durowoju, Olatunde S.Raysoni, Amit UDurowoju, OlatundeRazza, FrancescoDurstine, J LarryRebessi, FilippoDusse, LuciRecchia, Daniela R.Dutcher, DabrinaRedding, ColleenDuthie, MalcolmReddy, S. SurenderDuthie, Malcolm S.Redifer, JenniDutschke, GeorgRedlich, CarrieDutta, RahulRedmayne, MaryDwivedi, DipankarRedondo, GiselaDyer, FionaReed, Edward J.Dykstra-DeVette, Tiffany A.Reed, Beth GloverDysarz, TomaszReese, GerhardDyson, Yarneccia D.Rees-White, TristanDyson, PamelaReger, MichaelDzakpasu, MawuliRego, Rita De Cássia FrancoDziewit, LukaszRehman, LaureneE. Riley, KristenRehman, ShabnamEastin, Matthew D.Reibel, TracyEather, NarelleReich, JenniferEbeling, PeterReichel, Michael P.Ebersviller, SethReiches, MeredithEbisu, KeitaReichman, JeromeEby, LillianReid, St PatrickEckel, Sandrah P.Reid, Donald C.Economou, AthinaReifels, LennartEdelsbrunner, PeterReile, RainerEdgeston, AnnaReilly, RachelEdmondson, AmandaReiman, ArtoEdokpayi, Joshua NosaReimann, MareikeEdwards, MichaelReimer, ThordisEdwards, Christine AnnReineholm, CathrineEfird, JimmyReiner, MarkEfstratiou, Maria A.Reinhardt, Jan DEgawa, ShinichiReinhardt, Jan D.Egeland, Grace MReiss, BorisEggers, MariReiter, E. MirandaEggertsen, GostaRembiałkowska, EwaEgido, AngelRen, ZefangEhlen, J. ChristopherRen, QingzhongEide, ArneRen, HongyanEijkelkamp, BartRenard, Jean-BaptisteEisler, KarenResongles, EleonoreEker, SibelRestivo, VincenzoEkundayo, OlugbemigaRevuelta Iniesta, RaquelEkúndayò, Olúgbémiga T.Rey, LuisEkuni, DaisukeReyes-Sandoval, ArturoEl Ghoch, MarwanReynaga-Ornelas, LuxanaEl Shahawy, OmarReynolds, LoriElasra, AmiraRezaei-Gomari, SinaEleftheratos, KostasRezaie Boroon, Mohammad HassanEleftheriadis, TheodorosReznik, Jacqueline E.Elekes, ZsuzsannaRhee, GregElf, JessicaRibaldone, Davide GiuseppeElias, MiguelRibeiro, HelenaEllam, RobRibeiro, Ana IsabelEllen Posey, JenniferRibeiro, EdnaEllervik, ChristinaRibeiro, Rita Cláudia CardosoElliot, EsiRibeiro, JorgeElliott, BradleyRibeiro, OscarElliott, Michael B.Riccardo, OrusaEllis, CameronRiccio, AngeloElsafti, HishamRice, Penelope A.Elshall, Ahmed S.Rice, DavidEmerson, Dawn M.Rice, Robert H.Emilsson, LouiseRice, SamaraEmmanuel, EvensRichalet, Jean-PaulEmmer, AdamRichards, Sarah FieldEna, JavierRichards, JulieEndersby, RaeleneRichardson, DanielEndevelt, RonitRichardson, CliveEndriulaitienė, AuksėRichter, Jason P.Engan, Jon ArneRicking, MathiasEngdahl, BrianRickwood, DebraEnnis, EdelRider, G. NicEnoiu, RazvanRidgers, NickyEory, VeraRidinger, GarretEr, VanessaRiedel, BernhardErat, Anna MargaretaRiederer, AnneErazo, MarciaRiehle, MichaelErb, Klaus J.Rienecke, ReneeErbacher, Terri A.Rietra, ReneErbas, BircanRigante, DonatoEremeeva, MarinaRiggsbee, KristinErgler, ChristinaRigotti, ThomasErickson, RebeccaRiiser, AmundEriksson, CharliRikard, Robert VannErmler, SibylleRikkert, Marcel OldeErnis, SaracevicRiley, MalcolmErokhin, VasiliiRiley, KevinErreygers, GuidoRim, Kyung TaekErsen, AliRimaitis, KęstutisEscuder-Bueno, IgnacioRinaldo, NatasciaEsmaeili, BehzadRindone, CorradoEspejel, IleanaRinge, DagmarEspinosa, Hugo G.Rissanen, RitvaEspinosa-Reyes, GuillermoRitchie, Elspeth CameronEsposito, CiroRiveiro-Barciela, MarEsser, HelenRivera, VictorEstape, Estela S.Rivera, Jason D.Estevan, IsaacRiza, ElenaEsteves Da Silva, Joaquim C.G.Robards, FionaEun, Ki-SooRoberts, RebeccaEvans, MarleneRoberts, JenniferEvans, Josie M.M.Roberts, HannahEvans, Sarah FRoberts, AeliEvans, John RobertRobertson, KirstenEvens, AnneRobillard, AlyssaEveringham, Jo-AnneRobitaille, EricEwen, Heidi HRoccuzzo, SebastianaEwert, AlanRocha, HelenaExadaktylos, AristomenisRoche, AnnExley, ChristopherRockett, IanEze, Ikenna C.Rocklöv, JoacimEze, Ikenna CRöder, StefanEzzeddine, ZeinabRodgers, MarkFabbricatore, MariantoniettaRodrigo Comino, JesúsFabiani, RobertoRodrigues, EugénioFabiano, BrunoRodrigues, IsabelFabijańczyk, PiotrRodriguez, FernandoFactor-Litvak, PamRodríguez, Rocio BarriosFaehling, MartinRodríguez, JuanFagerstrom, KarlRodriguez Couto, SusanaFai A. Lo, KwongRodriguez-Bravo, BlancaFajardo, Val AndrewRodríguez-Díaz, ManuelFajardo-Bullón, FernandoRodríguez-López, PedroFajersztajn, LaisRodríguez-Lozano, Francisco JavierFalavigna, GretaRodríguez-Seijo, AndresFalco, AlessandraRodriguez-Villamizar, Laura AndreaFalcón, Raúl M.Rodziewicz, JoannaFalgenhauer, LindaRogerio Pinheiro, PlácidoFalk, MartinRogers, William PrattFalletti, LuigiRogers, JuanFalup-Pecurariu, OanaRogers, Ann E.Fan, ZhijinRogula-Kozlowska, WiolettaFan, BinRogula-Kozłowska, WiolettaFang, Guor-ChengRoh, ChanghyunFang, Wei-TaRohr, Annette C.Fang, JimRój, JustynaFanizzi, Francesco PaoloRojo-Álvarez, José LuisFappi, AlanRöllin, Halina B.Farah, Paolo DavideRom, OrenFarahani, AliRomano, MeganFareed, AmberRomano Spica, VincenzoFaro, JamieRomaszko, JerzyFarooq, MuhammadRomeo, StefaniaFarre, AlbertRomer, DanFarrimond, HannahRomero, InmaculadaFarris, AlishaRomero Martínez, ÁngelFarrish, Kenneth W.Romero-Martin, MacarenaFauchier, Angele CRominski, SarahFaúndes, AníbalRömling, CorneliaFaustini, AnnunziataRoncero, CarlosFaustman, ElaineRonchetti, SimonaFavalli, SilviaRonen, AvnerFavas, Paulo J.C.Ronnebaum, JulieFawkes, SallyRonzi, SaraFayaz, MohammadrezaRöösli, MartinFedock, GinaRoot, MartinFedorova, Olga V.Rootman, IrvingFedoseeva, SvetlanaRoque, FátimaFeinn, Richard S.Roques, Christian-FrançoisFeldman, Steven R.Rosa, Maria JoseFeleszko, WojciechRosa, Bruce A.Felgenhauer, TylerRosa, FlavioFelgueiras, HelenaRosa, PaoloFell, JamesRosales-Mayor, EdmundoFeltner, CynthiaRosário, RafaelaFemenias, PaulaRosborg, IngegerdFeng, ZhanchunRose, OlafFeng, Zhi ChaoRose, Roderick AFerdyn-Grygierek, JoannaRose, JennieFerguson, AlesiaRose, ArthurFernandes, Ana S.Rosemarin, ArnoFernandez, MarinaRosen, BarryFernandez, MarielaRosenberg, BethFernández, Julio BlancoRosenthal, MeaganFernández, MarioRoser, KatharinaFernández, LucíaRos-McDonnell, LorenzoFernández, BelénRosołowska-Huszcz, DanutaFernández-Bustos, Juan GregorioRospenda, KathleenFernández-García, MartaRoss, Kirstin E.Fernandez-Llatas, CarlosRoss, VictoriaFernandez-Morales, Francisco JesusRoss, Michael W.Fernández-Morilla, MonicaRoss, AllisonFernández-Solà, JoaquimRossiter, RachelFerrante, MargheritaRostasy, KevinFerraro, Pietro ManuelRothermund, EvaFerreira, MarceloRotondi, MarioFerreira, Carlos MiguelRott, EduardFerreira, Hugo Enrique JúnezRoubertoux, PierreFerreira De Serpa, Sandro NunoRouco, JoséFerrer-Cascales, Rosario I.Rougeau, KathrynFerrero, GiulianaRoura, MariaFerrero, MarcoRovers, Sanne F.E.Ferri, DeliaRowlands, AlexFerri, Giovanni MariaRowlinson, SteveFerro, MatteoRozance, Paul J.Ferronato, NavarroRóżańska, AnnaFerster, Colin JayRozema, AndreaFewkes, AlanRozema, KyleFialkiewicz, WieslawRozgonjuk, DmitriFiandra, LuisaRu, SushanFiebig, AndréRuano Ravina, AlbertoFiedler, Brian H.Rubin, Stuart H.Fife-Schaw, ChrisRubino, Federico MariaFigueras, AlbertRubinstein, EllenFilipe, PortelaRubio, Ramón G.Filippelli, GabrielRubio-Bellido, CarlosFilon, Francesca LareseRuda González, AlbertFinch, BryanRudd, RimaFine, James BurkeRüegg, JoelleFinkel, MadelonRuffino, BarbaraFinkelman, RobertRugel, EmilyFinkenauer, CatrinRuiz, MarioFinn, SymmaRuiz Rubio, LeireFinneran, KevinRuiz-Barquín, RobertoFiore, SilviaRuiz-Casares, MónicaFiorella, Kathryn JoanRuiz-Fernández, María DoloresFioretto, AntoniettaRuiz-García, AlejandroFiori, EnricoRuiz-Robledillo, NicolásFirlak, MelikeRumney, PeterFirmino, HoracioRuotsalainen, HeidiFischer, FlorianRupprecht, ChristophFischer, LeonieRupprich, MarcoFischer, MatthiasRusanowska, PaulinaFisher, EdwardRusciano, DarioFisher, KarenRusinek, DagmaraFisk, Malcolm J.Russell, ArmisteadFitzGerald, GerryRussell, DavidFitzgerald, EdwardRussell, JoFlack, JohnRusso, CarloFlack, Kyle D.Russo, Mario VincenzoFlacke, JohannesRutherfurd, IanFlanagan, NinaRuzsics, IstvánFlatau, AndrewRyabov, IgorFlecha, RamonRyan, BrendanFleischer, AnneRyan, MichaelFleming, Richard K.Ryan, Michael P.Flemming, Hans-CurtRyan, PeterFletcher, Nicola F.Ryan, Brid M.Fletcher, Teresa B.Ryan, Sadie J.Fleury, Marie JoséeRybak, JustynaFlexeder, ClaudiaRyde, GemmaFlood, StephenRydstedt, LeifFlorek, EwaRyoo, SungweonFlott, Elizabeth A.Sá, RosáliaFloyd, MyronSaavedra, ÁngelesFluegge, Kyle R.Saavedra, Jose M.Fogarty, TimothySabatier, ColetteFolmer, Robert L.Saboga Nunes, LuisFont, JosepSaccani, CesareFontaine-Bisson, BénédicteSacco, EmilioFonte, Carla Alexandra MartinsSacristán, DanielFoody, MairéadSádaba, CharoFoong, Rachel E.Saeedi, PouyaForaboschi, PaoloSaevarsdottir, SaedisFord, DawnSaez, MarcFord, AndrewSager, ManfredFormisano, AntonioSagone, ElisabettaFornés, JoanaSaha, SanjibForoughmand Araabi, HoomanSaha, SubrataForsell, YvonneSaha, DebasishFortgang, RebeccaSaha, Sushanta KumarFortin, ThomasSahm, Laura JaneFortune, NicolaSahoo, SasmitaForward, SonjaSahoo, Pabitra KumarFosas, DanielSaini, ShikhaFoster, K. R.Saisho, YoshifumiFoster, StephanieSajdakowska, MartaFotis, TheoSakamuri, Siva Sankara Vara PrasadFouché, JulienSalaheen, SerajusFouladkhah, AliyarSalamone, FrancescoFourtounas, CostasSalas, Lucas A.Foweather, LawrenceSalata, StefanoFox, Mary A.Salazar, Leslie RamosFox, Mary AvisSaldarriaga-Noreña, HugoFox, StephenSaleem, MuhammadFraccascia, LucaSalerno, FrancoFrade, João Manuel GraçaSales, ChristopherFradkin, ChrisSalinas-Pérez, José AlbertoFraile Ardanuy, José JesúsSallam, Mohamed F.Francesca, CalastriniSallis, AmandaFrancis, JoelSalm, Adrienne K.Francuz, TomaszSalmon, JohnFranklin, PeterSalmon, PaulFranz, EelcoSalonen, HeidiFranzen, DaveSalottolo, KristinFratianni, SimonaSalter, MichaelFrayne, BruceSaluk-Bijak, JoannaFrazer, KateSalvatore, MirellaFredianelli, LucaSalvi, DarioFreedman, JaneSalvia, RosannaFreeman, Michael D.Samadi, Sayyed AliFrei, ThomasSamara, TheanoFrias, JoãoSambamoorthi, UshaFriederic, KarinSamhan-Arias, AlejandroFriedlmeier, WolfgangSampaio, Alcínia Z.Friedrich, RainerSampaio, AnaFriger, MichaelSampath, RuwanFriso, ValeriaSampson, Natalie R.Fristedt, SofiSamuel-Nakamura, ChristineFritz, Megan L.San Jose, RobertoFröhlich, EleonoreSan Pedro Veledo, María BelénFröhlich-Gildhoff, KlausSanada, KiyoshiFrohne, TinaSanches, SandraFronczyk, JoannaSanchez, Isabel MartínezFrontistis, ZachariasSánchez Loyo, Luis MiguelFrossard, Jean-PierreSanchez Martinez, GerardoFruchon, SéverineSánchez Rojo, AlbertoFu, JianshuangSanchez-Flack, JenniferFu, JianSanchez-Oliver, Antonio JFu, QiangSander, LasseFuentes-Bargues, José LuisSanders, Nicholas J.Fuertes, ElaineSanders, TarenFuhrimann, SamuelSanders, KatherineFujibe, FumiakiSanders, JamesFujita, ToshiyukiSanderson, PeterFujiwara, KoichiSandholz, SimoneFukuda, YoshiharuSandle, TimFullér, RobertSanduzzi, AlessandroFung, Ivan Wing HongSandy, LarissaFurey, SineadSanftenberg, LindaFurler, JohnSanganyado, EdmondFurmańczyk, KonradSangelaji, BahramFuruta, MichikoSankar, PamelaFusco, GiulioSanmiquel Pera, LluisFuster, HéctorSansom, GarettFutcher, JulieSansoni, JulitaFüzéki, EszterSant, HimanshuGaardbo Kuhn, KatrinSantikarn, ChamaiparnGabel, ShirleySantilio, AngelaGabhainn, Saoirse NicSantoro, Paolo EmilioGábor, FöldváriSantoro, FrancescoGadd, HollySantos, Maria Teresa DosGaebler-Spira, DeborahSantos, AngelaGaeta, Maria GabriellaSantos, Pedro PintoGaitán, Adriana V.Santos, EduardoGajda, MaksymilianSantos, Maria PaulaGajpal, YuvrajSantos, MónicaGalante, FrancescoSantos, José MariaGalasinski, DariuszSantos, JuanGaleazzi, Gian MariaSantos, Rafael M.Galetti, MariclaSantos-Gallego, CarlosGalindo, Velia Carolina OsunaSañudo-Fontaneda, Luis A.Galindo, NuriaSan-Valero, PauGalinha, CatarinaSaotome, ChikakoGallegos-Carrillo, KatiaSarapultsev, AlexeyGaller, HerbertSaraux, AlainGalloway, AlisonSardaro, RuggieroGalloway, TraceySarkar, BiswajitGalvani, EmersonSarker, Altalf H.Galvão, João RafaelSarmento, Joaquim MirandaGalway, LindsaySarnyai, ZoltanGamble, Donald S.Sarrat, ClaireGamet-Payrastre, LaurenceSarria-Santamera, AntonioGammon, CatherineSarriegi, Jose MariGammon, DeedeSarubbo, Maria FiorellaGańczak, MariaSasagawa, MasaGandre, CoralieSasahara, ShinichiroGao, Jing-FengSasai, HiroyukiGao, NaiPingSas-Nowosielski, KrzysztofGao, Shiqian SherrySatarug, SoisungwanGao, WayneSatchidanand, NikhilGao, YongSathe, Gajanan JalindarnathGao, MingmingSato, YukitaGarbaciak, WiesławSattar, FarhaGarbaras, AndriusSaunders, Daniel G.Garcia, OscarSaurav, KumarGarcia, RosarioSaurina, CarmeGarcia, ValerieSautto, Giuseppe A.Garcia, JeanetteSavage, KayeGarcia, João Nuno Pinto MirandaSavion, NaphtaliGarcia, Gabriel James M.Savoie Roskos, MatejaGarcía, FernandoSawni, AnjuGarcía, GregorioSayed Picciani, Bruna LavinasGarcía, MónicaSayón-Orea, CarmenGarcía, AngelaScandurra, CristianoGarcía De La Vega, AlfonsoScanlan, JustinGarcia-Gasca, Silvia AlejandraScannell Bryan, MollyGarcía-Germán, SolScaringi, GianvitoGarcía-Goñi, ManuelScarpellini, SabinaGarcia-Leon, ManuelScarpi, EmanuelaGarcía-Sánchez, AsunciónSchablon, AnjaGardener, SamanthaSchauss, AlexanderGardner, KathrynSchechter, Julia CorwinGarg, Charu CScheepers, Paul T. J.Gargano, Lisa MScheffler, ChristianeGari, MerceScheid, JenniferGarmendia, Azurmendi IgnacioSchell, LawrenceGarner, BiancaSchelling, JörgGarrote, JulioSchenk, ToddGarssen, BertScherr, SebastianGartner, CoralSchiller, GeorgGarus-Pakowska, AnnaSchindler, SimonGasana, JanvierSchindler, StefanGascón, MireiaSchirmer, BastianGašević, DanijelaSchito, EvaGąska, AdamSchjøll, AlexanderGaskell, GeorgeSchlachter, SvenjaGasparatos, DionisiosSchlenstedt, ChristianGaspari, JacopoSchlieber, MarisaGast, RebeccaSchmalwieser, AloisGatica, GiovannaSchmeltz, Michael T.Gaudet, D.Schmid, ThomasGaudez, ClarisseSchmidlin, ThomasGaudiano, BrandonSchmidt, MichaelGauthier, Patrick T.Schmidt, RebeccaGavriletea, MariusSchmidt, Allison M.Gazda, AnnaSchmoeckel, JulianGazo, RadoSchneeweis, AdinaGázquez Linares, José J.Schneider, PetraGazzola, PatriziaSchneider-Luftman, DeborahGe, ErjiaSchnell, IzhakGe, CuiSchnuch, AxelGe, CuiSchoenaker, Danielle AJMGe, XiaodongSchoenfeld, DavidGearey, MaryScholz, JohannesGebel, ThomasSchomerus, GeorgGefeller, OlafSchönauer, RobertGehring, Tito A.Schout, GertGeise, GeoffreySchröder, DietrichGeneroso, Jaqueline Da SilvaSchroeder, PatrickGeores, MarthaSchullehner, JörgGeorgakellos, Dimitrios A.Schulz, Peter J.George, CindySchulz, ElkeGeorgian, BadicuSchulz, BurkhardGeorgios, GkikasSchuna, JohnGeraghty, PatrickSchütte, StefanieGeraldo, AndreiaSchwab, KeriGerard, NormaSchwartz, Carolyn E.Gerbino, Maria GraziaSchwartz, Rebecca M.Gerger, HeikeSchwartz, GaryGerken, TobiasSchwieren, ChristianeGerkowicz, AgnieszkaScoffield, JessicaGerrard, YsabelScott, StephanieGervase Iwu, ChuxScott, LloydGerwin, WernerScrine, ClairGerwing, JenniferSea, Mandy M.M.Geusens, Piet P. M. M.Seabi, JosephGhandhi, ShanazSearle, Samuel D.Ghani, FatimaSeaton, Sarah EGhani Zadeh, HosseinSebastiá Frasquet, María TeresaGHAZALI, Daniel AihamSeclen, Segundo NicolasGhazi-Nezami, FarnazSecondi, LucaGhelardi, EmiliaSedloev, TheophilGhilli, MatteoSee, Lai-ChuGhodake, GajananSeeman, Mary V.Gholami, MaryamSefton, JoEllenGholson, DrewSegrin, ChrisGhosh, AbhijitSegura Medina, PatriciaGhosh, SumitaŠeibokaitė, LauraGhosh, SumitSeifried-Dübon, TanjaGhulam, MustafaSeki, KatsutoshiGiaccone, ValerioSekine, YoshikaGiachello, Aida LuzSellers, SamuelGiacomino, AgneseSelosse, SandrineGianfagna, FrancescoSelvamani, AmuthaGiannarelli, StefaniaSelvarajan, RamganeshGianni, PesSelvaratnam, ThineshGiannopoulou, IfigeneiaSelya, ArielleGibbons, JeffSembajwe, GraceGibson, LauraSenent-Aparicio, JavierGiese, HelgeSengupta, AditiGigante, AntoniettaSenwo, ZacharyGijón Nogueron, GabrielSeo, YoungminGijs, MarliesSeoane, FernandoGilani, G. SarwarSeposo, XerxesGilbert, LucySepúlveda, MarcosGilbey, AndrewSera, FrancescoGilbride, Kimberley A.Serafini, GianlucaGiles, Luisa V.Serchi, TommasoGilkey, DaveSéré, GeoffroyGill, TiffanySerefko, AnnaGilliland, JasonSerio, FrancescaGilmour, StuartSerra-Moreno, RuthGil-Ortega, MartaSerrano, EduardoGiorgi, GabrieleSerrano, AntonioGiovene Di Girasole, EleonoraServidio, RoccoGiraldo, PilarServin, Argentina EGiri, SubhasisSestili, CristinaGirling, RobertSeth, RataneshGissler, MikaSetti, IlariaGitlow, LynnSetyan, AriGiuliano, MarinaSeyoum, WondwosenGiungato, PasqualeSgrigna, GregorioGiuntella, OseaSha, ZheGivehchi, RahelehShaban, Ramon Z.Gjestland, TrulsShack, LorraineGkemitzi, AlexandraShackelford, Rodney E.Głąbska, DominikaShah, Rachana D.Gładyszewska-Fiedoruk, KatarzynaShahab, LionGlattacker, ManuelaShahariar, ShayebGlavan, MatjažShahi, ShaileshGlavee-Geo, RichardShahid, RizwanGleason, Jessie A.Shahmoradi, MahdiGlowacz, AdamShakya, Kabindra ManGlynn, Peter J.Shaman, JeffreyGnudi, SaverioShamasunder, BhavnaGobattoni, FedericaShan, ChaoGobba, FabriziomariaShan, Xiaojun (Gene)Gobbo, MassimilianoShang, BaopingGobert, DeniseShankar, EswarGodebo, Tewodros R.Shannon, JerryGodoy-Vitorino, FilipaShao, TianjieGohlke, Julia M.Shao, QiangGolan, RachelSharafeldin, NohaGold, Laura S.Sharafeldin, TamerGoldman, Myla D.Sharer, MelissaGoldoni, MatteoSharifi, AyyoobGoldstein, Adam O.Sharifi, ErfanehGoldstein, SusanSharma, AshishGoldstein, Rachel RosenbergSharma, RohitGolomb, BeatriceSharma, UmakantGomes, Thayse NatachaSharma, SureshGómez Ciriano, Emilio JoséSharmin, SoniaGómez Piqueras, PedroShaw, Richard J.Gomez-Baya, DiegoShe, JiangfengGómez-Corona, CarlosShechter, AriGomez-Lobo, VeronicaSheehan, DianaGomez-Perez, Sandra L.Sheehan, Mary C.Gómez-Rey, PilarSheffield, PerryGomez-Salgado, JuanShelton, Chasity M.Gómez-Zorita, SaioaShen, JunGOMMES, René AlexShen, YaoGoncalves, OlivierShen, Chwan-LiGonçalves, GuilhermeShenoi, Asha N.Gonçalves, LuísaShepherd, DanielGong, ShiweiShepherd, AndrewGong, YuSherchan, SamendraGoñi, PilarSherer, NathanGoniewicz, KrzysztofShi, XueqingGonzalez Crespo, RubenShi, HaiyunGonzález Jiménez, EmilioShi, JishuGonzález Valero, GabrielShi, JingGonzalez-Barcala, Francisco JavierShi, JunboGonzalez-Casanova, InesShi, WenboGonzález-González, RogelioShi, HuahongGonzález-Navarro, PilarShi, XiaoyangGonzález-Rivera, Juan AníbalShi, YuanGonzález-Sosa, EnriqueShiao, S. Pamela K.Goodarzi, AaronShiau, Jenq-TzongGoodier, Martin R.Shibata, SeijiGoodla, LavanyaShieh, Dar-BinGoodman, PatShields, John PGoodwin, RobinShiffman, SaulGoodwin, LauraShih, PattiGoodwin, IanShil, NirajGoovaerts, PierreShimada, MasakoGorczynski, PaulShimek, Jo AnnaGordon, ChloeShin, Soo-YongGorgoglione, AngelaShin, Andrew C.Gorham, EdwardShin, Won SopGorini, GiuseppeShin, LauraGorini, FrancescaShin, Hyeong-MooGörlich, DennisShin, Hai-RimGorman, ShelleyShipman, KateGorman, RichShiramizu, BruceGorr, MatthewShiyanbola, Olayinka OGoswami, HaridhanShobha, PoudelGoto, KazuyaShoji, KotaroGou, ZhonghuaShokrollahi, PeymanGouget, BarbaraSholkamy, EmanGould, Dinah J.Short, LeonieGourbesville, PhilippeShrestha, VinishGouveia, Élvio RúbioShrestha, NarayanGowda, MadanShtrepi, LouenaGoyal, RajniShu, KeshengGozalo, Guillermo ReyShuang, QingGrabarczyk, MałgorzataShyr, Oliver F.Graber, JudithSiak Junpei, ElizabethGraber, KimSibanda, MelusiGracia, EnriqueSicakova, AlenaGradinaru, Andrei CristianSicard, PierreGradoni, LuigiSidani, JaimeGrafius, DarrenSiddique, ShabanaGragnano, AndreaSiddiqui, MuhammadGraham, Amanda L.Siebenhofer, MatthäusGranada, Camille E.Siebers, RobGranata, GiuseppeSiefken, KatjaGranata, FrancescoSiegel, Eric R.Grant, AirdreSiegel, Robert M.Grasser, Erik KonradSiegel, MartinGraveling, RichardSiegner, AlanaGraves, LeeSiesmaa, EmmaGraves, RichardSigler, JeffreyGray, LesleySigmund, ErikGray, AndrewSignorelli, CarloGray, DeborahSigurdardottir, SigrunGray, SelenaSigurvinsdottir, Rannveig S.Gray, RichardSikora, AgnieszkaGray, Andrew B.Sila-Nowicka, KatarzynaGray, Janet M.Silibello, CamilloGray, EmilieSillanpää, NoraGrecksch, KevinSilva, Gabriela VenturaGreen, NathanSilva, Analiza M.Greene, ClaireSilva, Denize Francisca DaGreenfield, ThomasSilva, Danilo Rodrigues Pereira DaGreenleaf, ChristySilva, Margarida F. B.Greenleaf, Arie T.Silva, Ana LuisaGregg, LucileSilva, Shamali DeGreim, HelmutSilva, Luiz Eduardo VirgilioGrieco, DomenicoSilva, LígiaGrieger, Jessica ASilver, Monica K.Griffin, TomSim, KyraGriffiths, EmmaSim, Chang SunGrischek, ThomasSimanek, AmandaGrishkan, IsabellaSimecka, Jerry W.Grochans, ElzbietaSimoes, PedroGrochtdreis, ThomasSimon, MatthewGroen, GunterSimoncini, KymGroffik, DorotaSimoni, JanGrossi, GiancarloSimons, Shellie R.Grossman, EllieSimons, KelseyGrossman, Jennifer M.Simpungwe, E.Growe, RoslinSims, JaneGruszecka-Kosowska, AgnieszkaSinden, KathrynGu, YianSingh, HarsimranGu, YuchaoSingh, GurdeepGu, FengSingh, AnuragGuagliano, JustinSingh, AjitGuan, Ng ChongSingh, ChanpreetGuariso, AndreaSingh, RajeshGuariso, GiorgioSingh, RupaliGudey, Shyam KumarSingla, Daisy R.Guell, CorneliaSingovszka, EvaGuenel, PascalSinha, SudarsonGuerra-Balic, MyriamSiniscalco, DarioGuerrero, Anthony P.Siobhan, WeareGuerrero Barona, EloisaSioen, GilesGuerriero, MassimoSips, PatrickGuevara, Pilar EgüezSiraj, Amir S.Gugerell, KatharinaSirsat, SujataGui, ZhipengSiskind, VictorGUIDA, MicheleSissung, Tristan M.Guidi, Caterina FrancescaSit, CindyGuilabert, MercedesSitnik-Warchulska, KatarzynaGuillén-Lambea, SilviaSixsmith, JudithGuimarães, Guilherme VeigaSjöblom, RolfGuina, JeffreySjoeholm, Karina KnudsmarkGuinness, JosephSkelly, ChrisGuirguis, KristenSki, ChantalGuirguis, AmiraSkilodimou, Hariklia D.Guldris, Secundino CigarranSklar, MarisaGulis, GabrielSkoczko, IwonaGulley, TaunaSkopeliti, AndrianiGulliver, PaulineSkórzyńska-Dziduszko, Katarzyna E.Gullón, PedroSkouloudis, Andreas N.Gump, BrooksSkrzypiec, GraceGundamaraju, RohitSlade, KarenGunn, LucySlaughter, JamesGunn, RachelSleet, DavidGunnarsdóttir, María J.Sloan, ChantelGunnarsdóttir, IngibjörgŚlusarska, BarbaraGunzler, Douglas DavidSly, TimothyGuo, YanyongSmalls, Britanny L.Guo, BrianSmani, YounesGuo, MingSmarandache, FlorentinGuo, LiangSmarr, Benjamin LGuo, Chun XiangSmit, Johannes HendrikusGupta, KajalSmith, MichaelGupta, VijayalaxmiSmith, HollieGUPTA, KULDEEPSmith, CarolGupta, NidhiSmith, Justin DeanGupta, BhawnaSmith, Leonard B.Gurung, AnobhaSmith, SianGurung, Thulo RamSmith, SandraGusiatin, MariuszSmith, BrandtGuski, RainerSmith, ClaireGustafson, AlisonSmith, Miichael (Mike)Gustafsson, SusanneSmułek, WojciechGustavsson, TorbjörnSnijder, MiekeGuthrie, RobertSnowden, TimothyGutierrez, KristieSoares, Mario J.Gutierrez, Peter M.Soares, HelenaGutiérrez, AaronSobisch, TitusGutiérrez, Jose De SolaSobocińska, MagdalenaGutiérrez, KristieSoenen, StijnGutleb, ArnoSogorb Sánchez, Miguel ÁngelGuxens, MònicaSohaib, OsamaGuzek, DominikaSojic, AleksandraGuzman, ChristianSokal-Gutierrez, KarenGyant, LaVerneSokas, RosemaryGyawali, PradipSokoloski, KevinHa, SandieSoles, GillianHa, HoehunSolís García Del Pozo, JuliánHaack, BarrySolomou, KaterinaHaapala-Biggs, IrjaSolovieva, YuliaHaarbauer-Krupa, JulietSolovyev, NikolayHaardoerfer, RegineSoltero, EricaHabib Perez, OlindaSoltysik, BartłomiejHabicht, JeanSone, HidekoHachiya, TsuyoshiSong, Min-SukHackett, DanielSong, JieHadgraft, Nyssa T.Song, RanranHadgraft, NyssaSong, SuyongHadjiiski, LubomirSong, MalinHadziabdic, EminaSong, YongzeHaex, BartSong, XianfengHagan, ChristopherSong, Melodie YunjuHagberg, MatsSong, FujianHaight, Joel M.Song, GeobooHaile, Tadele MeashoSong, HongqingHajigholizadeh, MohammadSonke, Jeroen E.Hakkenberg, ChristopherSönmez, SevilHale, BeverleySontag, MarciHall, JamesSopena, NievesHall, RalphSophie, WalshHall, AllysonSorahan, TomHall, Eric E.Sørensen, KristineHallen-Adams, HeatherSoria, JuanHall-Mendelin, SonjaSornay-Rendu, ElisabethHaluza, DanielaSosna, JustynaHama, SusumuSotunde, OlusolaHama, SarkawtSoudeyns, HugoHameed, ShaffaSoultos, Nikolaos D.Hamer, GabrielSoundy, AndyHamilton, KerrySousa, SofiaHamilton-Williams, EmmaSousa, Álvaro Francisco LopesHan, BingSousa, ArturoHan, JayoungSousa, NelsonHan, SeungYongSousa, Hélder SHan, Jeong-WonSousa, RitaHan, Seung JinSousan, SinanHan, LijianSouth, Robbie M.Han, QifengSouth, AndrewHanaeus, JörgenSowa-Kofta, AgnieszkaHandley, TonelleSpagnoli, PaolaHanes, DouglasSpagnuolo, ValeriaHanifzadeh, MohammadmatinSpalevic, VeliborHanisch, MarcelSpallone, RobertaHanke, WojciechSparke, MatthewHanna, Lezley-AnneSparkes, ValerieHanna, ElizabethSpear, Suzanne E.Hansen, Claus D.Speklé, Erwin M.Hansen, AlanaSpence, SueHansen, Jaron C.Sperlì, GiancarloHansmann, RalfSpiliotopoulos, MariosHanson, Karla L.Spillner, EdzardHanson, BuckSpinazze, AndreaHansson, KarinSpires, StevenHao, HaiguangSpittle, BruceHaque, ReinaSpitzenstetter, FlorenceHarada, KoujiSpizzichino, ValeriaHarada, KazuhiroSpreat, ScottHarbison, JustinSpringgate, BenHarder, MarieSprunger, JoelHarding, ScottSpurio, RobertoHardingham, Jennifer E.Spyros, GkelisHarduar Morano, LaurelSpyrou, ChristosHariharan, HarrySquires, PeterHaring, RobinSquires, VictorHarja, MariaSrivastava, PranayHarjumaa, MarjaSrour, Issam M.Harley, DavidSt Clair-Thompson, HelenHarmon, AlisonSt Hilaire, MelissaHarper, GordonSt Hill, CatherineHarrington, Daniel W.St.Helen, GideonHarrington, JulieStabile, LucaHarrington, Robert J.Stacchi, ClaudioHarrington, DierdreStack, StevenHarrington, Deirdre M.Stafford, GloriaHarris, Jason T.Stafford, FrankHarris, WilliamStakenborg, NathalieHarris, Ted D.Stalsberg, RagnaHarris, DeborahStamatis, NikolaosHarris, Joseph B.Stanca, Sarmiza ElenaHarrison, Lisa MStanciu, CameliaHarro, JaanusStanek, AgataHart, Joy L.Stanhope, JessicaHarth, VolkerStanistreet, DebbiHartmann, ChristinaStansfeld, StephenHaruna, SamuelStansfield, BenHarvey, AdamStark, Azadeh T.Harville, EmilyStarościak, WojciechHarzer, ClaudiaSteelman, VictoriaHasanagas, Nikolaos D.Steenkamp, MalindaHascher, TinaStefan, MariusHaschke, FerdinandStefanelli, PatriziaHashimoto, MasayukiStefani, AmbraHasmun, NorenStefanoff, PawelHass, AmirŠteff, MichalHassan, FauziyaSteffen, ArminHassan, Amr AbdulfattahStefler, DenesHassan, AmanySteidtmann, DanaHassan, AmrSteiger, AxelHassan, Sherif T. S.Stein, Robert M.Hassink, JanSteinberg, Julia R.Hata, Fernando TeruhikoSteiner, ZviHatchel, TylerStejskal, JanHaubenhofer, DoritSteliarova-Foucher, EvaHauck, Fern R.Stella, SimoneHaukka, KaisaStellman, StevenHauret, LaetitiaStendahl, OlleHausberger, StefanStenhuus, BoHauser, PeterStepnik, MaciejHawcroft, ClaireSterling, DavidHawkins, KeelyStevens, RebekahHawkins, MarquisStevenson, KathrynHayami, HitoshiStewart, LauraHayashi, RyujiStewart, Alex G.Haycraft, EmmaStewart, Orion TheodoreHayes, KatieStewart, Alan E.Hayward, R. DavidStewart Williams, JenniferHazell, PhilipSthel, Marcelo SilvaHazelton, PamelaStilianakis, NikolaosHe, YanlingStilianakis, Nikolaos I.He, MeiziStingone, JeanetteHe, KevinStock, GregoryHe, JunStockman, CarolineHe, CenlinStoker, AaronHe, AinaStokes, LynissaHeal, MathewStolt, MinnaHealy, William M.Stoneham, MelissaHearst, MaryStorcksdieck Genannt Bonsmann, StefanHeath, Gregory W.Stough, RogerHector, DebraStovner, Lars JacobHedman, LinnéaStowers, Kristen CookseyHege, AdamStrasser, RogerHegerl, UlrichStringfellow, Anne M.Heilmayr, DietlindeStroeve, SybertHeinen, MirjamStrohfus, Pamela K.Heinimann, HansStroshine, MeghanHeinonen-Tanski, HelviStroud, CraigHeinrich, JoachimStroud, JacquelineHeinze, KareenStrubelt, HenningHejduk, LeszekStrucinski, PawelHelbich, MarcoStrudwick, XantheHellström, LisaStuchlik, PatrickHelman, ShaunStudzińska-Sroka, ElżbietaHelmig, DetlevSturtevant, JoyHelmy, Yosra A.Stylianou, MichalisHemingway, AnnSu, ChunmingHemphill, Jean CroceSu, JunfengHenderson, RitaSuárez, RafaelHendricks, MarccusSubbaraman, Meenakshi S.Hendrikx, PascalSubbiah, MuruganHendryx, MichaelSubburaj, Anitha SarahHeng, YanSuda, KenichiHenley, Shauna C.Suder, AgnieszkaHennessy, DwightSudol, MiroslawHennessy, Emily AldenSudzina, FrantisekHenri, DominicSuen, I-ShianHenrickson, MarkSuggs, SuzanneHenriques, IsabelSugiyama, KemmyoHenry, LisaSuh, Dong HeeHenson, AdriannaSui, ZhixianHeo, SeulkeeSukumaran, Sunil KumarHeo, JongbaeSukumaran, AnilHer, YoungguSulaiman, Ahmad Hatim BinHerbert, AnthonySullivan, David W.Herckes, PierreSullivan, KimberlyHerman, KrzysztofSummers, Robert J.Hermoso-Orzáez, Manuel JesúsSummers, James KevinHernan, AmandaSumner, WaltonHernandez, LadiaSun, ChaynHernandez, QuetzalcoatlSun, XiaoqiangHernández, AdrianaSun, Guan-ChengHernandez-Tejada, MelbaSun, HongyongHerold, David M.Sun, NaweiHerr, Raphael M.Sun, GuohuiHerrera, ManuelSun, YanHerrera-May, AgustínSun, XuanHerrera-Melián, José AlbertoSun, XiaojieHerrero, María JesúsSun, ShengzhiHerrmann, AlinaSun, Feng-HuaHessels, AmandaSun, HongweiHetrick, Sarah ElisabethSun, Kai SingHettiarachchi, HiroshanSundar, ReshmaHeuss-Aßbichler, SorayaSundqvist, Jan-OlovHewlings, Susan J.Sundram, FrederickHibbert, KathleenSuner, SelimHickey, HuhanaSuñer Soler, Maria RosaHickey, MarthaSung, KyungeunHida, AzumiSuppes, LauraHidalgo, MarcoSurdacka, AnnaHigueras, Pablo L.Sutherland, JohnHildreth, BlakeSuzan-Monti, MarieHill, KimberleySvanemyr, JoarHill, HollySvartengren, MagnusHill, JennieSwango-Wilson, AmyHill, Elaine L.Swann, Brian JeffreyHill, EthanSwaroop, MamtaHill, EdwardSwendeman, DallasHiller, Amie L.Sy, AngelaHillier-Brown, Frances C.Sy, IbrahimaHillman, AubreySybilski, Adam J.Hime, Neil J.Symons, MartynHimoto, TakashiSzablewski, LeszekHines, Erin PiasSzabó, SzilárdHinkelmann, ReinhardSzabó Nagy, AndreaHinyard, Leslie J.Szafranek, AnnaHirai, ItaruSzafron, MichaelHirano, Y.Szczepaniak, WłodzimierzHiratsuka, MasahiroSzeiffova Bacova, BarbaraHirose, YuichiSzekely, GyorgyHiscock, RosemarySzékely, AndreaHjorth, PederSzeląg, MaciejHlaing, WayWaySzepietowska, Ewa MałgorzataHo, K. F.Szeto, GraceHo, Yuh-ShanSzlagatys-Sidorkiewicz, AgnieszkaHo, RogerSzóstak, MariuszHo, CyrusSzumny, Antoni JacekHoang, PhuSzychowski, JeffHoare, ErinSzymańska-Pulikowska, AgataHobbs, MatthewSzyszkowicz, MieczyslawHodge, AllisonSzyszkowicz, MieczysławHodge, VernonT. Zaleski, RosemaryHodges, LynetteTada, AkioHodgson, IanTadanaga, KiyoharuHoek, GerardTadashi, ToyamaHoekstra, TrynkeTaeihagh, ArazHoene, MiriamTagg, AlexanderHoff, RodneyTaghizadeh, NiloofarHoffman, StevenTaherdangkoo, RezaHoffmann, Ellen M.Taheri, ShimaHofman-Caris, RobertaTai, Bik-Wai BilvickHöger, RainerTajan, NicolasHogg-Johnson, SheilahTajani, FrancescoHoghooghi, NahalTakahashi, SatoruHogue, Candace M.Takahashi, KenHohwü, LenaTakakai, FumiakiHokama, TomikoTakaki, ManabuHoła, BozenaTakala, JukkaHolford, Theodore RTakano, TomomiHolgado Ramos, DanielTakebayashi, HidekiHolland, Stephen M.Takegata, MizukiHolle, RolfTakenaka, ShotaHolley, ClareTalati, ArdesheerHolliday, DAWNTalbot, BenoitHolm, RochelleTalbott, EvelynHolmes-Rovner, MargaretTaliaferro, LindsayHolmner, ÅsaTallini, MarcoHolttum, SueTamošaitienė, JolantaHolvad, TorbenTamulevicius, NaurisHomaeigohar, ShahinTan, XiaoHomma, TetsuyaTan, JingHon, CarolTan, Chun LiangHonda, ShunichiTan, QingHoneycutt, MichaelTanabe, MasakiHong, PeiyingTanaka, NobuhikoHong, JiangtaoTanaka, ShinsukeHong, Young-SeoubTanaka, JunkoHongisto, ValtteriTandra, Pavan KumarHönig, VáclavTang, JingyinHonn, Kimberly A.Tang, NelsonHopkins, NicolaTang, YouhuaHoque, ShamiaTang, QiyiHoracek, TanyaTang, JianjunHoran, KristinTang, XiaochenHori, HajimeTanigawa, TetsuyaHoriuchi, MasahiroTanner, GritHoriuchi, SatoshiTao, YuHorn, JohannesTao, FengmingHorton, TeresaTao, LimingHorwitz, PierreTappia, ElenaHoshiko, HeatherTarazona-Santabalbina, Francisco JoséHoshiko, SumiTarman, KustiariyahHosick, PeterTarrant, MarieHosohata, KeikoTartakovsky, LeonidHossain, IqbalTartari, GianniHossain, Md EkhtearTarver, MichelleHosseini, MohsenTasmin, SairaHou, JunTaverna, LiviaHou, Wen-HsuanTavolaro, AdalgisaHouck, Philip DTavoschi, LaraHouck, James A.Taylor, JoeHoudmont, JonathanTaylor, Henry LouisHoward, JeffreyTaylor, TimHoward, NatashaTaylor, GeoffreyHoward, GuyTaylor, AdamHowden-Chapman, PhilippaTaylor, JenniferHowe, Amanda CarolineTaylor, MatthewHowe, StephanieTchangani, Ayeley P.Høyer-Kruse, JensTeismann, TobiasHribar, LawrenceTei-Tominaga, MakiHroncová, EmíliaTeixeira, MónicaHseu, Zeng-YeiTeixeira, LaetitiaHsieh, Huey-HongTelesca, VitoHsieh, Vivian Chia-RongTelford, Rohan M.Hsieh, Yi - PingTemple, NormanHsieh, Nan-HungTemple, LizHsieh, Yi -FangTemple, JeromeyHsieh, Ching-HuaTendero, MarjorieHsieh, Wen-ChuanTeng, Ru-JengHsu, Chia-YuehTenkate, ThomasHsu, Shao YiuTeron, LemirHsu, Hui-ChuanTerzano, KathrynHsu, Ming-FuTeske, JenniferHsu, Li-ChunTestoni, InesHsu, Sung-PoTettegah, SharonHsu, Yea-JenTeuber, RamonaHtun, Htet LinThai, PhongHu, YujieTham, RachelHu, HelenThavorn, KednapaHu, WeiThelin, EricHu, XuefengTheodorsson, Gudjon ElvarHu, GuoqingTheorell, ToresHu, HuiTheregowda, RanjaniHu, Dong GuiThibodeaux, TerryHuan, Tzung-ChengThielens, ArnoHuang, Wendy YajunThom, DominikHuang, KaiThomas, JacquelineHuang, Wu-JangThomas, Roger E.Huang, DanlianThomas, ReubenHuang, HongtaiThomas, EwanHuang, Yu-TungThomas, GeraldineHuang, Yan-JangThomas, Stephanie MargareteHuang, Cheng-LiangThomas, TerryHuang, XiaoyanThomée, SaraHuang, XudongThompson, ClaireHuang, GanlinThomsen, HaukeHuang, Chun-JungThomson, Rachel M.Huang, ChaoThornley, SimonHuang, Guan-JhongThornton, ThereseHuang, HaiyanThorsteinsson, EinarHuang, Xiao (Eric)Tiatia-Seath, JemaimaHubková, BeátaTibério, Iolanda De Fátima Lopes CalvoHudda, NeelakshiTigbe, WilliamHudson, IreneTigkas, DimitrisHudson, StantonTilak, Amey S.Huestis, Samantha E.Tilley, ElizabethHugelius, KarinTimmins, KateHughes, JenniferTinnerberg, HåkanHughes, Jean R.Tippett, VivienneHughes, Matthew WilliamTiraboschi, MicheleHui, XuanTiranti, DavideHui, Bryant P HTischler, DirkHui-Ching, WuTitze, SylviaHuijser, HenkTkavc, RokHulsegge, GerbenTobin, KatyHummel, KarinToderi, StefanoHumphreys, CathyTodeschini, SaraHung, Mei-ChuanToelle, BrettHung, Li-FangToft, GunnarHung, YungTogo, FumiharuHung, Yung-TseToh, Li SheanHunter, RuthTomaszewska, EwaHunter, Elizabeth G.Tomata, YasutakeHuntington-Moskos, LuzTomczak, AndrzejHuq, NazmulTomioka, MichiyoHurley, JamesTommasino, LuigiHurley, RachelTomporowski, Phillip D.Hursthouse, AndrewToms, Leisa-MareeHurtubia, RicardoTonacci, AlessandroHuss, FredrikTong, ZhemingHussain, IftikharTong, TammyHuxley, PeterTong, RuipengHuybregts, LievenTonolo, GiancarloHwang, AmieToo, Lay SanHwang, Su-LunToomey, ClodaghHylander, Lars D.Topa, GabrielaHyun, EunjungTopala, IonutHyysalo, SampsaToporowska, MagdalenaIannace, GinoTordera, NúriaIatsenko, IgorTorem, Mauricio LeonardoIbarra-Mejia, GabrielTorgerson, ChelseyIbiebele, IbinaboToronto, ColeenIbrahim, Mohamed BabikerTorpy, FraserIbrahim Salem, SalemTorre, CarmeloIchihashi, MasamitsuTorres, João PauloIde, KazukiTorres López, M IsabelIdler, Ellen L.Torres Vaamonde, Jose EnriqueIdowu Ajayi, AnthonyTorres-Ortega, SaúlIfelebuegu, AugustineTorrey, William C.Ifelebuegu, Augustine OsamorTortini, RiccardoIgoe, DamienToselli, StefaniaIguchi, TaisenTotaro, MicheleIhara, Emily S.Tóth-Bodrogi, EditIizuka, KatsumiTouil-Boukoffa, ChafiaIkegaya, NaokiToumba, MeropiIkehata, KeisukeTovilla-Zárate, Carlos AlfonsoIl’yasova, DoraTovino, Stacey AnnIlluminati, GiulioTownsend, JulieIlluminati, SilviaTrabace, LuigiaIlmakunnas, PekkaTran, Binh Q.Imamura, FumiakiTranfo, GiovannaImeson, AntonTrematerra, PasqualeImmler, UlliTremolada, MartaIncalzi, Raffaele AntonelliTrencher, GregIngusci, EmanuelaTrevisan, AndreaInnes, AntheaTrif, MonicaInniss, Enos C.Triunfo, StefaniaInohara, TakehiroTrögl, JosefInoue, ShigeruTrovato, FrankInoue, RyoTrue, LarissaInoue, AtsukoTruman, PennyInsenser, MaríaTsagarakis, KonstantinosInvernizzi, MarcoTsagkari, ErifyliInvitto, SaraTsai, Wei-LunIoelovich, Michael YaTsai, Wen-HsienIossifidou, EleniTsai, Tzu-IIpsakis, DimitrisTsai, CarrieIqbal, Hafiz M.N.Tsakiris, Ioannis N.Irannezhad, MasoudTsatsaris, AndreasIreland, Patrick R.Tseles, DimitriosIrga, PeterTsesmelis, DemetriosIrvine, IanTsiamis, CostasIsaacs, AntonTsikouras, PanagiotisIsaacson, MichalTsiros, MargaritaIsgor, ZeynepTso, For YueIshihara, ToruTsow, Francis (Tsing)Ishtiaq, KhandkerTsuda, ToshihideIsola, GaetanoTsukiyama-Kohara, KyokoItahashi, SyuichiTucker, RaymondItalia, SalvatoreTuells, JoséItkonen, TiinaTulkens, PaulIto, TomokoTuluri, FrancisIttenbach, Richard F.Tulve, NicolleIuliano, SandraTunaru, SorinIvan Ramírez, JorgeTundys, BlankaIvany, Colonel ChristopherTungal, RichaIvascu, LarisaTuomisto, JoukoIvey, CesunicaTupėnaitė, LauraIwu, EmiliaTurgeon, NathalieIzawa, Kazuhiro P.Turiel, MaurizioIzenstark, Dina MarieTurki, SadokIzydorczyk, BernardettaTurkmen, MutluIzzi, ValerioTurnbull, PaulineJablonka, AlexandraTurnbull, DavidJablonska, EwaTurner, Barbara J.Jackowska, MartaTurner, BenjaminJackson, Chandra L.Turner, JoannaJackson, Alun C.Turner, BrianJackson, SarahTurunen, AnuJackson, SaraTutak, MagdalenaJacob, BenjaminTuuri, GeorgiannaJacob III, PeytonTwisk, JosJacobs, David E.Twiss, Michael R.Jacobs, MiriamTwumasi, RicardoJacobs, KarenTwyman, LauraJacobsen, RamuneTykkyläinen, MarkkuJacobsen, Geir WenbergTyler, Kimberly A.Jacobsohn, LiaTyrrell, EdwardJafari, RahelehTyson, JulianJagannathan, RamTytła, MalwinaJäger, PiaTzaphlidou, MargarethJahns, LisaTzavara, CharaJaine, RichardTzivian, LilianaJaiswal, MamtaUang, RandyJaja, IshmaelUcar, ZennureJajtner, AdamUchmanowicz, IzabellaJakab, GergelyUchôa, Carlos Henrique GomesJakóbczyk-Karpierz, SabinaUddin, ShahadatJakubowicz, AndrewUeda, KayoJalava, KatriUehara, IwaoJalayondeja, ChutimaUfnal, MarcinJames, CaroleUgarte, María DoloresJames, Katherine A.Ugrina, MarinJames, StevenUjihara, MasakiJames, PeterUkrainczyk, NevenJames, Shirley A.Ulhoi, JohnJampani, MaheshUlibarri, GerardJanczukowicz, WojciechUlrich, CraigJang, Jiun-HueiUm, Ki-HyunJang, SiwonUmanzor, ScheryJanicki, GrzegorzUnchwaniwala, NuruddinJanko, MarkUnderhill, ElsaJankovic, MomciloUnlu, IsikJankowska, MartaUnni, Elizabeth J.Jansen, JesseUnwin, PeterJanssen, Ellen MargreetUpadhyay, AbhinavJanssen, SarahUphoff, NoortjeJanssen, SabineUpshur, Carole CJaradat, Raed MUrasinska-Wojcik, BarbaraJarcuska, PeterUrban, AlešJarosz-Chobot, PrzemysławaUrban, NoelJasienska, GrazynaUrien, NastassiaJaved, Arshad AliUrra, José MiguelJavid, Roxana J.Urrestarazu, ElenaJay, Sarah M.Useche, SergioJay, NaomiUsman, MuhammadJayarathne, ThilinaUstaoglu, EdaJego-Sablier, MaevaUstinaviciene, RūtaJekova, IrenaUusi-Rasi, KirstiJelalian, ElissaVaarno, JenniJelski, WojciechVaezghasemi, MasoudJena, Prasant KumarVagero, DennyJenkins, Jill A.Vaiciunas, TomasJennings, BruceVaismoradi, MojtabaJennings, GeorgeValdivia, PedroJensen, RogerVale, PauloJensen, Eric D.Valentin, Melissa M.Jentschke, MatthiasValentine, Sarah EJeon, Gyeong-sukValentukeviciene, MarinaJeong, Tae-YongValenzuela, RodrigoJeong, BongjuVallamsundar, SuriyaJeong, MichelleVallely, LisaJeronimus, Bertus F.Vallianatos, FilipposJesola, EmmanuelVallini, GiovanniJeter, Cameron B.Vallone, DonnaJetter, James J.Valsero, María HijosaJeuring, JelmerValvi, DaniaJi, JohnVan Dam, PieterJI, YuelongVan Den Ende, JefJi, HongVan Den Heuvel, Ellen GHMJi, YingboVan Den Putte, BasJia, WeiVan Der Ende, Peter. C.Jia, Zhongweivan der Molen, Henk FJiang, HengVan Der Voordt, TheoJiang, WayneVan Dijk, JitseJiang, WenbinVan Doorn, RobertJiang, FengVan Eerd, DwayneJiang, JingzeVan Eyk, HelenJiang, JingVan Ginneken, EstherJianu, IuliaVan Kamp, IreneJiao, LiminVan Kann, Dave (D.H.H.)Jiao, JunfengVan Kann, DaveJim, LukVan Lieshout, LisetteJimenez, PaulVan Meeteren, MichielJimenez, EmilioVan Nassau, FemkeJimenez, ΒlancaVan Osch, Frits H. M.Jiménez-Ballesta, RaimundoVAN RUIJVEN, BasJin, JianjunVan Spijker, BregjeJin, LiVan Wagoner, Nicholas J.Jin, AndrewVan Wyk, PaulaJin, LeiVance, TerrenceJin, XinfangVance, MarinaJindal, CharulataVandenberg, Ann E.Jin-Ding, LinVanderloo, Leigh M.Jitaru, PetruVanderslott, SamanthaJoanna, ChwaszczVanos, JenniferJohansson, Michael A.Vanscheeuwijck, PatrickJohn, DeborahVanwambeke, SophieJohner, RandyVárbíró, GáborJohnsen, Stig OleVarela Torres, Jorge JavierJohnson, Amy K.Vargiu, EloisaJohnson, SarahVarju, CeciliaJohnson, Reed F.Varotsos, CostasJohnson, DaynaVarrica, DanielaJohnson, NicholasVasconcelos, JoãoJohnson, RutherfordVasconcelos Pinto, MartaJohnson-Motoyama, MichelleVasiliades, LamprosJohnston, Kelly L.Vasko, VasylJokinen, KimmoVásquez, ElizabethJonek-Kowalska, IzabelaVassiliadis, ThemistoklisJones, Rena R.Vaughn, AllisonJones, Rodney P.Vavilis, DimitriosJones, TiffanyVaz Moreira, IvoneJones, MalcolmVaz-Moreira, IvoneJones, AndrewVazou, SpyridoulaJones, Jeffery A.Vázquez Duhalt, RafaelJones, Michael L.Vázquez Lara, Juana MaríaJones, Kelly K.Vázquez Sánchez, DanielJones-Eversley, SharonVazquez-Alaniz, FernandoJonk, YvonneVedal, SverreJonsson, KRVedøy, Tord FinneJordao, LuisaVeeranki, S PhaniJorens, Philippe G.Veerman, J LennertJørgensen, RikkeVega-Zamora, ManuelaJoshi, AnupVeiga, Feliciano HenriquesJoshi, Pushpa RajVelasco Garrido, MarcialJoyner, AndrewVelimirovic-Fanfani, MilicaJu, Lu-KwangVelíšek, JosefJukić Peladić, NikolinaVemula, HarikaJukkala, TanyaVenables, DanJulian, TimVenäläinen, SallaJung, JiyeonVenkataraman, KartikJung, Se-YounVenkatesh, SiddarthJung, KyujinVentura, AlisonJunne, FlorianVenturini, ElisaJurzik, LarsVenus, JoachimJust, NathalieVerani, MarcoJyothi, Rajesh KumarVerbruggen, MarijkeKabir, RussellVerd, SergioKabir, GolamVergara, Lizandra Garcia LupiKaczynski, AndrewVerger, PhilippeKadotani, HiroshiVerloigne, MaïtéKagawa, TakahideVerma, ArjunKageshima, YosukeVersino, ElisabettaKahan, DavidVesper, StephenKahler, David M.Vetreno, Ryan P.Kahler, DavidVetter, SylviaKahn, MichaelVetter, Stefan W.Kai, MichiakiVicente, Manuel Sastre DeKairuz, ThereseVicente-Vicente, José LuisKaklamanou, DaphneVidal, TâniaKaklauskas, ArturasVidal, AlixKalambura, SanjaVidoni, Eric D.Kalantzi, Olga-IoannaViegas, SusanaKalbarczyk, RobertViegas, CarlaKalber, TammyVieneau, DanielleKalderis, DimitriosVignali, ValeriaKaleta, DorotaVignolio, OsvaldoKalhan, SatishViguié, CatherineKalinich, JohnVijayakumar, SarathKalkstein, AdamVilaro, Melissa J.Källén, BengtVilaro, MelissaKalmar, FerencVillalonga-Olives, EsterKambezidis, Harry D.Villani, MichelaKamdem, Jean PaulVillanti, Andrea C.Kamen, Charles S.Villa-Parra, Ana CeciliaKamenov, KaloyanVillarreal, BrandilynnKamisalic, AidaVillarreal Molina, Maris TeresaKampas, AthanasiosVillotti, PatriziaKanamori, MasaoVimercati, LuigiKanavillil, NandakumarVincent, GraceKanayochukwu Nduka, JohnVinceti, MarcoKandel, Devi RamVingada, José Vítor De SousaKandel, SarojVinjé, JanKandul, SerhiyVink, MarkKane, EleanorViola, SerenaKang, Eun HaVioque, JesusKang, JaekuVirginio Agostoni, CarloKang, SanghoonVirkki, TuijaKang, YoungcheolVisentin, ChiaraKang, Soo JungVisser, BartKang, Choong-MinVisser, AnitaKang, ZihoViswanadam, GouthamKang, SinyoungVitacca, MicheleKanning, MartinaVitali, MatteoKansal, MonikaVithana, ChamindraKantamaneni, KomaliVitry, AgnesKantono, KevinVivat, BellaKao, Nien-HsinVives Cases, CarmenKapalavavi, BrahmamVliet, SaraKapferer-Seebacher, InesVoaklander, Donald C.Kaplan, SigalVogels, ChantalKaplan, Bonnie J.Voliani, ValerioKaplinski, OlegVolken, ThomasKaplowitz, Stan A.Vollaard, NielsKaposztasova, DanielaVollmann, ManjaKappell, AnthonyVollmer, RachelKarabina, Sonia-AthinaVolpe, RichardKarakhan, AliVon Szombathely, MalteKaramagioli, EvikaVona, MarcoKaranis, PanagiotisVonk, RoelKarczmarczyk, AgnieszkaVonk, Roel J.Karim, NasiaraVoPham, TrangKarimian, NiloofarVorhees, DonnaKärkkäinen, SirpaVossos, HeleneKarmakar, ParthaVosvick, Mark A.Karol, ElizabethVoudris, VassilisKarpowicz, JolantaVoutos, YorghosKarppinen, AriVozza, IoleKarras, SpyridonVulchanova, Mila DimitrovaKarymbalis, EfthimiosVuorio, AlpoKasai, ShinyaWachs, SebastianKasai, NaoyaWachter, Jan K.Kasaraneni, Varun K.Wachtler, CarolineKashiwagi, ShinichiroWackowski, OliviaKaškonienė, VilmaWada, KeikoKasneci, EnkelejdaWade, Charles E.Kasparian, JérômeWadsworth, Danielle D.Kastaun, SabrinaWaersted, MortenKastrin, AndrejWagenfeld, Amy E.Kasule, Omar HasanWagg, AdrianKasztelan, ArmandWagner, Norbert L.Katada, KazuhiroWagner, DaleKataoka, TomoyaWagner-Hartl, VerenaKatapally, TarunWagnild, JanelleKate, HarveyWahiduzzaman, MDKatsantonis, DimitriosWaibel, ChristianKatsikas, StamatiosWainwright, NaldaKatsikini, MariaWakabayashi, IchiroKattelmann, KendraWalcek, ChrisKatuchova, JanaWałdykowski, PiotrKatz, Daniel J.Walker, Ana PaulaKatz, DavidWalker, Blake ByronKaucic, MassimilianoWalker, MarvinKauffman, Kathryn MarieWall, L LewisKaufman, PamelaWallace, Joshua S.Kauppinen, AriWallace, KedraKawai, Vivian K.Wallace, DavidKawai, ToshiyukiWallace-Bell, MarkKawamura, TomoyukiWaller, Lance A.Kawano, KouichiroWallor, EvelynKaye, LindaWalls, Kelvin L.Kayetha, Vinay K.Walrath, Christine M.Kayser, LarsWalsh, ShanaKayser, KlausWalsh, RobertKazakis, NerantzisWalters, GarethKazanas, StephanieWalters, DianneKazimírová, MariaWalton, AnnMarie L.Ke, YinghaiWalton-Moss, Benita JeanneKeck, MargretWan Mohd Yunus, Wan Mohd AzamKecojevic, VladislavWanchai, AusaneeKędzierska, EwaWang, ChengKędzierska-Mieszkowska, SabinaWang, RebeccaKeen, PatriciaWang, YichuanKeesing, SharonWang, ShengruiKeida, ElizabethWang, JianqiangKeidar, OsnatWang, YangKeil, ThomasWang, ZheKeiler, Annekathrin MartinaWang, Kuan-YuanKeinan-Boker, LitalWang, ShigongKeinonen, TuulaWang, Grace YKeler, AndreasWang, QuanyingKelishadi, RoyaWang, JianKelley, RachaelWang, Jian-WenKelly, Terri-AnnWang, Xiao-HuaKelly, PatrickWang, ChengjinKelly, KimberlyWang, DengjunKemper, Han CGWang, ChenghaoKenkel, DonaldWang, DaoyuanKennedy, JonathanWang, JunKenntner- Mabiala, RamonaWang, MengKenny, NatashaWang, FeiKent, BrianneWang, Fu-KwunKent, KatherineWang, ZhijunKephart, WesleyWang, Y. KenKeraminiyage, KaushalWang, ZhiKeramydas, ChristosWang, YulingKeshteli, Ammar HassanzadehWang, EndongKessler, RodgerWang, XiaoqinKessler, Theresa A.Wang, ZhenboKett, MariaWang, YuKeynan, IritWang, RuoniuKhamkhash, AibyekWang, Ching-HoKhan, Usman TWang, ShaohuaKhan, MahmoodWang, JunboKhanna, SunaliWang, HuanKharrazi, AliWang, Harry H.X.Khemka, GauravWang, XuKhorasani, Elham SahebkarWang, KeshengKhouri Miraglia, Simone Georges ElWang, ZenengKhubchandani, JagdishWang, YangjunKia, Seyed MostafaWang, KarynKibbe, AlexandraWang, Meng–TingKicińska, AlicjaWang, Teresa W.Kidd, Lisa A.Wang, QunKieffer, NicolasWang, Huei-JuKiely, MaireadWang, YouxinKillikelly, ClareWang, YingKim, JarimWang, MingHuaKim, ChoonsigWang, QiKim, HyungWang, YiKim, YoungdeokWang, KimberleyKim, Dong-KyunWang, BinKim, SeoyongWang, Chiu-YenKim, Woo-JinWang, JianxuKim, HyeongsuWang, Feng-ShengKim, SojungWang, JinfengKim, NickWanigatunga, Amal AsiriKim, Chang-GilWard, Kenneth D.Kim, Chun-BaeWard, JeremyKim, JunghoonWard, EmmaKim, Sun-YoungWarden, Stuart J.Kim, Jeong HeeWarfield, Marji EricksonKim, Seong DaeWark, StuartKim, DohyeongWarner, DougKim, Bianca Sung MiWarner, Lisa MarieKim, HyunWarren, ScottKim, HyungKyooWarriner, KeithKim, DohyoungWarszyński, PiotrKim, JayWarwell, Marcus V.Kim, George R.Wase, NishikantKim, Hyoung-RyoulWasfi, Rania A.Kim, MinguWashbourne, Carla-LeanneKim, GunwooWashburn, JasonKim, Young-JiWashburn, David J.Kim, Yeon-HaWąsik, JacekKim, Myungseob (Edward)Watanabe, ToruKim, Jung WookWaterlot, ChristopheKim, Henry M.Waters, MatthewKim, ChyerWaters, CatherineKim, SoyeonWatkins, JuliaKimber-Trojnar, ŻanetaWatkins, Daphne C.Kimbrough, David EugeneWątróbski, JarosławKim-Mozeleski, Jin EWattage, PremachandraKimoto, ShigeruWawrzyńczak-Szaban, AnnaKimura, AkioWdowiak, ArturKimura, Aya HWeaver, ScottKinaciyan, TamarWebb, Cameron E.Kindler, JosephWebb, KarenKindu, MengistieWebb, CameronKing, Jessica L.Weber, SilvanaKingshott, BrianWeber, KonradinKingston, JohnWeber, Ellerie SKinnunen, Jaana M.Webster, Noah J.Kinsky, Suzanne MWebster, Craig StephenKintziger, KristinaWeeks, FaithKinzel, CarolinaWęgrzyński, WojciechKirby, RussellWei, XuebinKirchengast, SylviaWei, BeiganKirk, SaraWei, YonglongKirken, Robert A.Wei, GuiwuKirkøen, BenedicteWei, Yehua DennisKirst, HenningWei, XuanKistler, KurtWeierstall, RolandKistler, MagdalenaWeigand, HaraldKita, SachikoWeigelt, OliverKiyono, KenWeigl, MatthiasKjellgren, AnetteWeijs, LiesbethKjellstrom, TordWeiner, JudithKlebe, SonjaWeiner, Shira SchecterKleiman, Evan M.Weinmann, TobiasKlein, PenelopeWeir, DawnKleinert, EvelynWeismayer, ChristianKlemeš, Jiří JaromírWelch, DavidKlimkowicz-Pawlas, AgnieszkaWelgamage Don, AakashKlostermann, ClaudiaWells, E. ChristianKlotz, Stephen A.Wells, EllenKlukowska-Rötzler, JolantaWelz, JulianeKlusmann, VerenaWen, SijinKnapstad, MaritWen, BeiKneepkens, C. M. FrankWenos, JeanneKnell, GregoryWertz, Philip W.Knight, HelenWesseler, JustusKnighton, ShaninaWessels, IngaKnoblauch, Astrid M.Wesson, DonKnott, RachelWest, JeffreyKnott, PatrickWest, ChristopherKnox, EmilyWesterholm, PeterKnudsen, LisbethWettstein, GalKnutsen, Helle KatrineWeyermann, MariaKo, Young DaeWheeler, DavidKo, Jane L.Wheeler, AnneKobayashi, HiromitsuWhite, JenniferKobayashi, TakeshiWhite, JanineKobayashi, YumiWhite, Brandi M.Kocatepe, AyberkWhite, Helen K.Kociu, ArbenWhiting, TerryKocot, JoannaWhitlock, JanisKoda, EugeniuszWhitney, JeanKodama, HideyaWhittaker, KarenKodoudakis, NikosWhittaker, PeterKoehler, ManfredWhitworth, KristinaKoga, Patrick MariusWibig, JoannaKohler, JefferyWiblin, JessicaKohler, Lindsay N.Wibowo, SantosoKohli, RichieWichrowski, Matthew J.Kohn, MaryWickliffe, JeffreyKoizumi, AkioWidziewicz, KamilaKokado, KentaWiech, PawelKolarik, Andrew J.Wielgomas, BartoszKolerski, TomaszWiens, KirstenKolluru, GopiWierzejska, ReginaKolmes, Steven A.Wilburn, Kathleen M.Kolo, JerryWildner, ManfredKomives, TamasWilkinson, StephenKomljenovic, DraganWillard, SuzanneKomori, TeruhisaWilliam, LauraKonarski, JanWilliams, Janet F.Kondo, MichelleWilliams, Edith M.Kondo, KayokoWilliams, Jessica A. R.Kones, RichardWilliams, Joah L.Konradsen, HanneWillis, DonKonstantakopoulou, EvgeniaWilson, Mark J.Konstantinidou, ValentiniWilson, Fernando A.Kontoni, Denise-PenelopeWilson, LeighKoohsari, Mohammad JavadWilson, PeterKool, BridgetWilson, Debra RoseKoop, StefWilson, James G.Koorey, GlenWilson, MaxwelKopeć, MichałWilts, HennningKopsaftis, ZoeWincenciak, JoannaKorajkic, AsjaWinchester, LeeKorcz, AgataWindapo, Abimbola OlukemiKorhonen, IlkkaWinijkul, EkbordinKornprat, PeterWink, RüdigerKoronczai, BeatrixWinkler, MirkoKorpela, KatriWinkvist, AnnaKorte, CassandraWinn, Deborah M.Kortelainen, TerttuWinneke, GerhardKorzeniewska, EwaWinters, StephenKósa, KarolinaWinwood, PeterKosarac, IvanaWipfli, BradKosari, SamWitcher, AnthonyKoshiyama, MasafumiWithanachchi, Sisira S.Kosinski, ErykWitt, MartinKosteli, Maria ChristinaWittlich, MarcKostka, TomaszWłodarczyk-Marciniak, RenataKostoglou, MargaritisWojciechowski, AdamKothgassner, Oswald D.Wójkowska-Mach, JadwigaKou, GangWojnicki, IgorKousha, TermehWolff, KristinaKoutelidakis, Antonios E.Wolkoff, PederKoutropoulos, ApostolosWolny, LidiaKovacic, Melinda ButschWolter, IlkaKovanen, Petri T.Wong, PaulinaKowal, MałgorzataWong, Chi-kin KenchiKowalczewski, ChristineWong, WilliamKowalska, MalgorzataWong, James Tsz FungKowalska-Góralska, MonikaWong, Ho Ting FrankKoyama, AsukaWong, Ling TimKozak, AnnaWong, Stephen H.S.Kozlov, MikhailWONG, Ngai Sze CandyKragovic, MilanWoo, May K.Krajcsák, ZoltánWoo, Jong MinKrajewska-Włodarczyk, MagdalenaWood, CarlyKrajewski, PiotrWood, Lincoln C.Kramer, LouisaWood, JenKratzke, CynthiaWoodfield, LorayneKraus De Camargo, OlafWoods-Chabane, Gwen C.Kraut, AllenWoolway, Richard IestynKrefis, Anne CarolineWothge, JördisKreider, RichardWright, Charlotte M.Krems, JaimieWright, Elizabeth QuinnKreuzinger, NorbertWright-Berryman, JenniferKreuzpointner, LudwigWu, HongKrieger, Jean-PhilippeWu, LiyunKrienert, Jessie L.Wu, JianyongKrishna, JyotiWu, WeiningKristensen, TroelsWu, QiKristensen, Louise JaneWu, ZhifengKristman, Vicki L.Wu, LingtaoKról, AleksanderWu, BingKropf, JohannesWu, FanKrüger, CarstenWu, HongzhuanKrysinska, KarolinaWu, SaoweiKrystofiak, TomaszWu, FenKrystofik, MarkWu, YupengKrzyścin, JanuszWu, WenchenKu, SeockmoWunderlich, Shahla M.Kuan, Wen-HuiWurl, JobstKubena, KarenWurtele, Sandy K.Kubota, KeiichiWyczarska-Kokot, JoannaKuca, KamilWyndow, PaulaKucharska-Newton, AnnaWynne, OliviaKudielka, Brigitte M.Wyver, ShirleyKudláček, MichalXia, YankaiKuehl, SilkeXia, BoKuhnert, MatthiasXia, YuchenKuipers, AbeleXiang, YutaoKujawski, WojciechXiang, JianjunKulak, WojciechXiang, YuKumar, AmitXiao, RongKumar, NitinXiao, YanKumar, GauravXiaoli, AlusKumar, BinodXie, HuixiangKumari, AshaXie, XiaopingKundi, MichaelXie, HualinKunst, Anton E.Xiong, YongzhuKuo, Shu-lungXu, IrisKuo, Meng-IXu, FurongKuo, TonyXu, JuanKuorwel, KuorwelXu, ZhiweiKurata, MieXu, LuKurdowska, Anna K.Xu, YuanKurdve, MartinXu, LiKurihara, OsamuXu, ChengdongKurihara, NobutakaXu, XunKuriqi, AlbanXu, Elvis GenboKurmi, OmXu, ZhihengKuroda, YujiroXu, JingKurti, Allison N.Xu, BiaoKusch-Brandt, SigridXu, LinKutschke, SabineXu, JianhuaKuwabara, MasanariXu, YunhongKuwahara, KeisukeXue, BaowenKvaal, KariXue, BingKwak, Kyung-HwanXuerui, GaoKwiecień, IwonaYael Wright, CaradeeKwok, RichardYager, EricKwon, SoyoungYamada, SatoruKwon, JayminYamaguchi, NobuyasuKyne, DeanYamakawa-Kobayashi, KimikoKyriakopoulos, GrigoriosYamamoto, NaomichiLa, ThuyYamato, Tiê ParmaLa Parra-Casado, DanielYamatsu, KojiLa Torre, GiuseppeYan, ShengminLaatikainen, TiinaYan, JPLabouliere, ChristaYanar, BasakLac, AndrewYanda, MuraliLachenmeier, DirkYang, Rong-SenLachmann, BerndYang, LinshengLadino, Luis AntonioYang, Wei-hsiungLafforgue, MichelYang, FanLåftman, Sara BrolinYang, YongLaganà, PasqualinaYang, XiaomeiLahart, IanYang, KaiteLahiri, SriyankaYang, XiaolinLai, Peggy S.Yang, HongshunLai, Shih-WeiYang, BoyiLai, IvanYang, QinghuaLai, LimingYang, XiaoLAI, Lufanna Ching-hanYang, LianpingLai, Chia-HsiangYang, ZhaojunLai, GavinYang, LingLaidlaw, MarkYano, ShozoLaird, YvonneYano, TakashiLaird, IanYao, YuChunLakhan, RamYao, JieLaking, GeorgeYared, RamiLaliotis, IoannisYates, Nathanael J.Lallana, María JesúsYau, YungLallier, Thomas E.Yavitt, JosephLam, JuleenYe, ShujunLam, Cho Kwong CharlieYeckel, Catherine WeikartLam, Simon C.Yeh, Ching-ChiangLam, Otto Lok TaoYeh, Li-TingLama, GyaneshYeh, Hsuan-HsienLamba, JasmeetYeh, Hsiao-PuLambert, Sharon F.Yeh, MayLambrechts, WimYela, Daniela AngerameLamina, ClaudiaYen, Cheng-FangLan, Cheng-CheYenerall, JackieLan, PingYetemen, OmerLancee, JaapYeung, Kam LeungLand, Kenneth C.Yifeng, ChenLand, Mary-AnneYiin, Lih-MingLandry, Michel D.Yin, JiananLane-Serff, GregoryYin, HongbinLang, JessicaYing, XiaohuaLange, Sabine S.Yliniemi, JuhoLange, JoannaYli-Tuomi, TarjaLange, EwaYllo, Kersti AliceLangford, Aisha T.Yokohira, MasanaoLangford, MitchelYokoo, HirokiLangiano, ElisaYokoshima, ShigenoriLangley, RickYongsi, Blaise NguendoLanglois, Peter H.Yoo, EnkiLanot, AntoineYoo, Do GuenLantz, FrédéricYoo, SeunghyunLanzinha, JoaoYoon, KayLanzola, GiordanoYoshiki, ShujiLaprise, MartineYoshinaga, JunLar, UriahYoshitake, HideakiLaraqui, Chakib El HoussineYoukee, DanielLarkin, Daniel J.Young, SeanLarkin, CelineYoung, Sean G.Larose, TriciaYoung, JeanineLarson, LincolnYoussofzadeh, VahabLärstad, MonaYu, XiaosongLasekan, John B.Yu, HaofeiLashley, MaryYu, RubyLassnig, HeimoYu, Shih-HengLasson, ElliotYu, HaoLászló, MakraYu, Tsung-HsienLatkowska, MonikaYu, Ignatius Tak-SunLau, Patrick W.C.Yu, BailangLau, Ho Yin EricYu, Chia-PinLauerma, HannuYu, Cheng-PingLaughter, BennieYue, XiaohangLaureano-Rosario, Abdiel E.Yue, YaojieLauret, PierreYuen, John WmLauretani, FulvioYumoto, HiromichiLauridsen, Jørgen TrankjærYunus, FakirLauritano, DorinaYurekli, AydaLauterbach, JohnZaborskis, ApolinarasLavandier, CatherineZabucchi, GiulianoLavender, AndrewZaccaroni, AnnalisaLaverack, GlennZacharias, JohnLavia, LisaZacharof, MyrtoLavigne, Salme E.Zadeh, Rana SaghaLavkulich, Les M.Zaffina, SalvatoreLavrencic, LouiseZagarins, SofijaLaw, Chi-kinZaharia, CarmenLaw, DavidZahnd, WhitneyLawn, SharonZahodne, LauraLawrence-Wood, EllieZahoor, HafizLawson, JustinZajda, JosephLayden, BrianneZakaria, KhalidLazard, AllisonŻakowska-Biemans, SylwiaLe, HuongZakrocka, IzabelaLeach, JonathanZalewska, MagdalenaLeak, TasharaZaman, Quazi Mohd Mahtab-uzLeal, WalterZandonadi, Renata PuppinLecerf, Jean-michelZanoli, LucaLeclercq, AlexandreZanzer, Yoghatama CindyaLedda, CaterinaZapolski, TamikaLedingham, MariekeZarfel, Gernot E.Lee, Ya-WenZarogoulidis, PaulLee, Chia-HwaZarone, FernandoLee, PetronaZavadskas, Edmundas KazimierasLee, PeterZavala, Gerardo A.Lee, PatriciaZavattaro, ElisaLee, JaeZawadzki, JarosławLee, KwonhoZeeb, HajoLee, HyunjungZeiner, MichaelaLee, Eun YoungZema, Demetrio AntonioLee, Wallis A.Zeman, Daniel MeierLee, Yung-JaanZeng, HuipingLee, Jae-YoungZeng, BoLee, JieunZeng, XiaoweiLee, JaeyoungZeng, YujinLee, Youn OkZeng, NanLee, Hae KookZeng, GuangmingLee, KennethZeng, shihongLee, SeungyeonZeng, HongmeiLee, Regina L. T.Zeng, HuangLee, Yuh-MingZetterström-Dahlqvist, HeléneLee, Pyoung-JikZgaga, LinaLee, Kuo-KauZhai, YujiaLee, JosephZhamsueva, Galina S.Lee, JuyoungZhan, XinminLee, Jae EunZhang, XueleiLee, Stacy H.N.Zhang, XiyuanLee, Jen-YaoZhang, ChunyongLee, Rebecca E.Zhang, DahaiLee, Yee MunZhang, YongLee, Joon KyuZhang, LeimingLee, HyokiZhang, YuanLee, Joe - MingZhang, YiLee, Hian KeeZhang, BaihaiLefering, RolfZhang, GuanshiLeffler, JonatanZhang, MelvynLegates, RichardZhang, YongpingLeger, ElsaZhang, PengLehman, Peggy W.Zhang, DingmeiLehnert, Lukas W.Zhang, WeiLeijs, Marike M.Zhang, TingruLeipner, JörgZhang, Rita PeihuaLeira, ManelZhang, GuoqingLeisner, JørgenZhang, CharlieLeitão, AnaZhang, DongLeitão, JoãoZhang, BaoqingLeitao-Alexandre, SaraZhang, ZengqiangLeitch, CarolZhang, JinLeiter, MichaelZhang, DongfengLelijveld, NatashaZhang, XiangLeMay, SteveZhang, LeiLemiere, CatherineZhang, TaoLemke, BrunoZhang, JianLeng, Xiaoyan IrisZhang, XinLenton, GavinZhang, YuanzhiLeo, Carlo GiacomoZhang, ZhengqiangLeón, Gerardo A.Zhao, YaqianLeón Pérez, Jose MariaZhao, ZhuohuiLeon Rubio, Jose MariaZhao, YongfengLeonard, PhilipZhao, BaoyinLeonard, AnneZhao, MinjuanLeonardi, GiovanniZhao, QiranLeone, Matthew C.Zheng, Yu-JunLeone, AntonioZheng, YuanrunLeonhard, ChristophZhou, XujuanLeppänen, Matti T.Zhou, Shang-MingLépy, ÉliseZhou, RuijieLera, María JoséZhou, LuxiLerario, AntonellaZhou, LifengLercher, PeterZhou, YingLerma, ClaudiaZhou, YibiaoLerner, HenrietteZhou, PeilingLessard, LauraZhou, SuhongLesser, IrisZhou, HuaiyuLeurs, KoenZhou, ZhijunLevene, LouisZhou, ZhiqinLeventhall, GeoffZhou, YubinLevesque, LucieZhu, LinLewin, EyalZhu, ZhenduoLewińska, KarolinaZhu, GuoqiangLewis, Joanne M.Zhuang, PingLewis, KathyZia, KashifLewis, DanielZiegler, JaneLewkowski, Jarosław A.Ziegler, Stephen J.Li, ZijianZielinski-Gutierrez, EmilyLi, Xiangyu (Dale)Ziemann, EwaLi, XuemeiZiemba, PawełLi, RuiZienius, DainiusLi, WenyiZikos, DimitriosLi, JingZikova, NadezdaLi, WeijuanZimmer, DavidLi, Shi ZhongZimmerman, EmilyLi, JessieZinszer, KateLi, Shu GengZiolkowska, JadLi, Hua-BinZiuzina, DanaLi, Kin-KitZlatko, NikoloskiLi, YingZodrow, Katherine R.Li, LongZolin, AnnaLi, ShunpingZou, LeiLi, YingruŹróbek-Sokolnik, AnnaLi, XiaodongZscheischler, JanaLi, DapengZubair, OpeyemiLi, KelinZubelzu Minguez, SergioLi, BoZumstein-Shaha, MayaLi, PingZuniga-Teran, AdrianaLi, MilingZurita Ortega, FélixLi, JinkeZurriaga, ÓscarLi, QingZUSMAN, EricLi, ChangZwahlen, MarcelLi, HaoZwoździak, AnnaLI, GuangdiŻyromski, AndrzejLi, ManyuZyśk, Janusz

